# Umbilical cord milking and delayed cord clamping for the prevention of neonatal hypoglycaemia: a systematic review and meta-analysis

**DOI:** 10.1186/s12884-024-06427-w

**Published:** 2024-04-08

**Authors:** Estelle D. Watson, Lily F Roberts, Jane E Harding, Caroline A Crowther, Luling Lin

**Affiliations:** Liggins Institute, The University of Auckland, 85 Park Road, Grafton, Auckland, 1023 New Zealand

**Keywords:** Neonatal hypoglycaemia, Delayed cord clamping, Umbilical cord milking, Placental transfusion

## Abstract

**Background:**

Placental management strategies such as umbilical cord milking and delayed cord clamping may provide a range of benefits for the newborn. The aim of this review was to assess the effectiveness of umbilical cord milking and delayed cord clamping for the prevention of neonatal hypoglycaemia.

**Methods:**

Three databases and five clinical trial registries were systematically reviewed to identify randomised controlled trials comparing umbilical cord milking or delayed cord clamping with control in term and preterm infants. The primary outcome was neonatal hypoglycaemia (study defined). Two independent reviewers conducted screening, data extraction and quality assessment. Quality of the included studies was assessed using the Cochrane Risk of Bias tool (RoB-2). Certainty of evidence was assessed using the Grading of Recommendations, Assessment, Development and Evaluation (GRADE) approach. Meta-analysis using a random effect model was done using Review Manager 5.4. The review was registered prospectively on PROSPERO (CRD42022356553).

**Results:**

Data from 71 studies and 14 268 infants were included in this review; 22 (2 537 infants) compared umbilical cord milking with control, and 50 studies (11 731 infants) compared delayed with early cord clamping. For umbilical cord milking there were no data on neonatal hypoglycaemia, and no differences between groups for any of the secondary outcomes. We found no evidence that delayed cord clamping reduced the incidence of hypoglycaemia (6 studies, 444 infants, RR = 0.87, CI: 0.58 to 1.30, *p* = 0.49, I^2^ = 0%). Delayed cord clamping was associated with a 27% reduction in neonatal mortality (15 studies, 3 041 infants, RR = 0.73, CI: 0.55 to 0.98, *p* = 0.03, I^2^ = 0%). We found no evidence for the effect of delayed cord clamping for any of the other outcomes. The certainty of evidence was low for all outcomes.

**Conclusion:**

We found no data for the effectiveness of umbilical cord milking on neonatal hypoglycaemia, and no evidence that delayed cord clamping reduced the incidence of hypoglycaemia, but the certainty of the evidence was low.

**Supplementary Information:**

The online version contains supplementary material available at 10.1186/s12884-024-06427-w.

## Background

Neonatal hypoglycaemia is one of the most common issues encountered in neonatal care. It occurs in 5–10% of healthy term infants [1], and in 27–54% of at-risk infants [2–4]. Severe or persistent low glucose concentrations have shown to negatively affect neurological development [2]. Therefore, strategies to prevent neonatal hypoglycaemia warrant investigation [5].

Waiting to clamp and cut the umbilical cord after the birth allows time for the transfer of blood in the placenta to the infant [6]. Delayed cord clamping (DCC) has been shown to provide a variety of short- and long-term benefits for the infant. These include increased neonatal haemoglobin concentrations, decreased incidence of intraventricular haemorrhage (IVH) [7], prevention of hypotension, increased Apgar scores and decreased mortality [8–11]. In preterm infants, DCC may reduce the risk of infant death by 27%, compared to early cord clamping (ECC) [11]. Its unsurprising, therefore, that the World Health Organisation and American College of Obstetricians and Gynaecologists recommend DCC (> 1 min after birth) for improved infant health [12, 13].

Umbilical cord milking (UCM) involves squeezing the umbilical cord several times from the placental end towards the infant [14, 15]. Since this technique can be completed quickly, it can provide an alternative placental transfusion in infants where DCC may be clinically inappropriate [14]. A review by Basile et al. [16] which included randomised controlled trials (RCTs) as well as other study designs, showed that UCM may be comparable to DCC in its effect on haematological parameters. Two recent systematic reviews including only RCTs also found that UCM is comparable to DCC in improving short term haematological outcomes in babies ≥ 34 weeks gestation [15, 17]. In preterm infants, 2 g/dL higher initial levels of haemoglobin have been found in the UCM group compared to ECC or DCC groups [18], and there is some low quality evidence that UCM may improve developmental outcomes when compared to DCC in preterm infants [19]. There appears to be no difference in risk of mortality for preterm babies receiving UCM compared to other cord management strategies [18], although the safety of UCM in extremely preterm infants remains unclear [20].

Once the cord is clamped and placental blood supply ceases, the newborn must adjust from dependence on their mother for fuel to initiating endogenous glucose production [21, 22]. Failure to adapt to this sudden interruption of glucose supply when the cord is clamped is the most common reason for neonatal hypoglycaemia [5, 23]. Placental transfusion through DCC or UCM provides extra blood and may potentially help protect against hypoglycaemia, but there is a paucity of information on this. Therefore, our objective was to perform a systematic review of the effects of DCC and UCM on the incidence of neonatal hypoglycaemia in both term and preterm infants.

## Methods

This review was conducted by following the methodology outlined in the Cochrane Handbook for Systematic Reviews of Interventions [24] and is reported following the Preferred Reporting Items for Systematic Reviews and Meta-analysis (PRISMA) guidelines [25]. This review was registered with the international database for prospective register of systematic reviews (PROSPERO) (ID: CRD42022356553).

### Search strategy and selection criteria

We searched MEDLINE (Ovid), Embase (Ovid), CINAHL Plus, the Cochrane Central Register of Controlled Trials (CENTRAL), Current Controlled Trials (www.controlled-trials.com), Clinical Trials (www.ClinicalTrials.gov), Australian and New Zealand Clinical Trials Registry (www.anzctr.org.au), and WHO ICTRP Search Portal (https://apps.who.int/trialsearch/), from inception until March 2023 (Appendix 1). Search results were imported into Covidence software [26] where titles and abstracts were independently screened for eligibility by two authors (EW,LR). Any disagreement was resolved by discussion or with a third author (LL). References of included studies were also screened for inclusion.

Inclusion criteria were term and preterm infants who underwent DCC (≥ 30 s, or study defined) compared to a control intervention (ECC, < 30 s or study defined) or UCM compared to a control intervention (other cord management strategies including ECC and DCC). We included published and unpublished RCTs without restrictions on language and publication date. Exclusion criteria included studies of only non-vigorous infants, or only those requiring resuscitation at birth. The eligibility of the studies was not based on reported outcomes.

The primary outcome was neonatal hypoglycaemia (study defined) before hospital discharge. Secondary outcomes were receipt of treatment for hypoglycaemia during initial hospital stay, number of episodes of hypoglycaemia during initial hospital stay, severity of hypoglycaemia (study defined), admission to special care nursery or neonatal intensive care unit (NICU), admission to special care nursery or NICU for hypoglycaemia, hypoglycaemic injury on brain imaging, blood glucose concentration during initial hospital stay, breastfeeding (study defined) at discharge, neurodevelopmental impairment (study defined), neonatal mortality, length of hospital stay, cost of intervention (as measured by study), and cost of neonatal care (as measured by study).

### Data extraction

Data were extracted by two authors (EW, LR) using a custom-designed form on Covidence. Data extracted included study design, location, year of publication, population, intervention details, and information relating to control, participant baseline, outcomes, and subgroups. Any discrepancies in extracted data were resolved by consensus. Risk of bias for all outcomes was independently assessed by two authors (EW, LR) using the Cochrane Risk of Bias (RoB-2) tool [24, 27]. Any disagreements were resolved by consensus, and if necessary, by discussion with a third review author (LL).

### Statistical analysis

Meta-analysis was undertaken separately for UCM and DCC using Review Manager 5.4.1. using random effect models [28]. For dichotomous outcomes, the risk ratios (RR) with 95% confidence intervals (CIs) were calculated. For continuous outcomes, the mean differences (MD) with 95% CIs were calculated. All data using median values (range or interquartile range) were converted to mean and standard deviation (SD) using the method of Wan et al. [29]. All glucose concentrations were converted to mmol/l.

The variability in effect estimates due to heterogeneity was determined by calculating the I^2^ and X^2^ for each analysis. Publication bias was determined by visual inspection of funnel plots, plotting the study effect size against the sample size, if there were enough studies (10 or more RCTs). If asymmetry was apparent, possible reasons were discussed. Direction of the findings tables were used to summarise the evidence if meta-analysis was not possible.

Planned subgroup analyses were: (1) Duration of delay before cord clamping (30–60 s vs > 60 s); (2) Gestational age (term vs preterm); (3) Mode of delivery (vaginal vs caesarean); (4) Birth setting (hospital vs non-hospital); (5) Maternal diabetes status (yes/no); (6) Babies at risk of hypoglycaemia (yes/no).

The Grading of Recommendations Assessment, Development and Evaluation (GRADE) approach [30] was used to assess the certainty of evidence for the following outcomes: (1) Neonatal hypoglycaemia (study defined); (2) Receipt of treatment for hypoglycaemia (study defined); (3) Severity of hypoglycaemia (study defined); (4) Admission to NICU for hypoglycaemia; (5) Length of initial hospital stay; (6) Breastfeeding (study defined) at hospital discharge.

## Results

### Search results

**Fig. 1:**
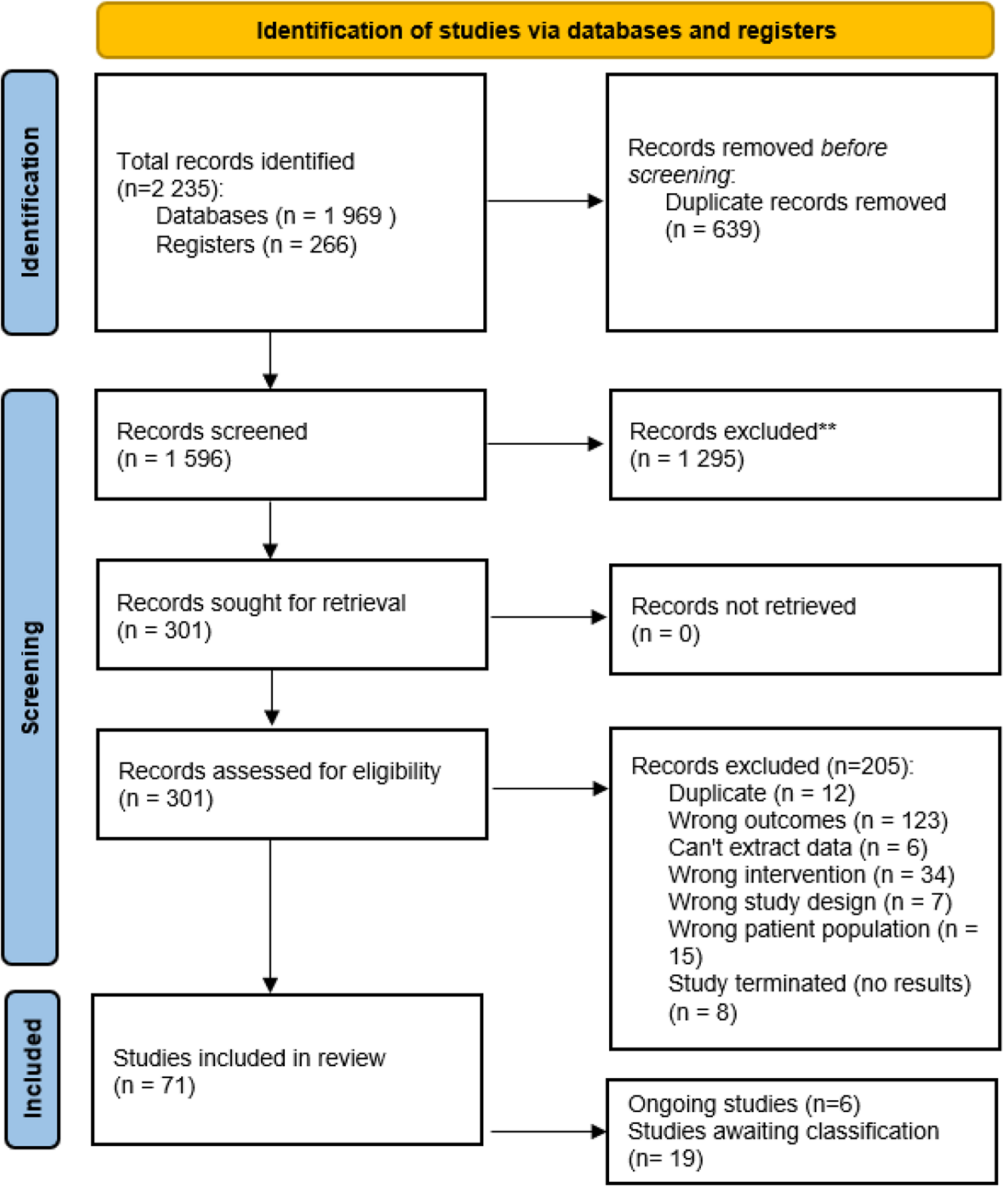
PRISMA flow diagram of search process

The initial search identified 2 235 potential records, of which 1 596 were screened after duplicates were removed and 301 full texts were assessed for eligibility. Full text screening excluded 209 records. A total of 92 studies were included in the review, with data from 71 studies included in the final analysis (Fig. 1). Authors of ongoing/unpublished studies were contacted to request current status or trial data but no unpublished data were available for inclusion.

### Characteristics of included studies

There were 21 studies assessing UCM compared to control, 16 of which included preterm infants. Fourteen of these studies (67%) were conducted in high income countries, 3 in upper-middle income countries, and 1 in lower-middle income countries [31] (Table 1). They were conducted between 2007 and 2022, and sample size ranged from 24 to 253 infants.

**Table 1:** Study characteristics

No.	Author/Year	Country	Participants	Participants, *n*	Intervention / Timing	Control / Timing	Outcomes
**Umbilical cord milking**							
1	Atia 2022	Saudi Arabia	Inclusion: preterm (24.0–34.6 weeks), singleton Exclusion: multifetal pregnancy, diagnosed congenital anomalies, fetal anaemia, considerable antepartum haemorrhage, category III cardiotocography tracing	200 (Intervention: 100, Control:100)	Cord was milked 4–5 times, at 10 cm/s	Cord was clamped at 45-60 s	Length of hospital stay, neonatal mortality
2	Chellappan 2022	India	Inclusion: preterm (27 – 32 weeks) Exclusion: monochorionic diamniotic twins, intrauterine growth restriction, hydrops fetalis, major congenital anomalies	179 (Intervention: 93; Control: 86)	Cord was milked 3 times for 10-20 s	Early cord clamping (undefined)	Length of hospital stay, neurodevelopmental outcomes at 6–12 months corrected age (Moderate to severe disability), neurological examination, Trivandrum development screening chart; Developmental assessment scale for Indian infants
3	Elimian 2014	USA	Inclusion: singleton, preterm (24 – 34.0 weeks) Exclusion: major fetal structural or chromosomal abnormalities, multiple gestations, maternal diabetes, intrauterine growth restriction, non-reassuring fetal heart tracings	200 (Intervention: 99; Control: 101)	Cord was clamped after 30 s, and milked 3–4 times	Cord was clamped within 5 s of birth	Neonatal mortality
4	El-Naggar 2022	Canada	Inclusion: singleton, preterm (24—30.6 weeks) Exclusion: monochorionic twins, major congenital anomalies, placental abruption, fetal anaemia, intention to withhold resuscitation	65 (Intervention:34; Control: 31)	Cord was milked three times, speed 10 cm/s	Cord was clamped within 10 s of birth	Neurodevelopmental outcomes at 36 months corrected age (Bayley Scales of Infant and Toddler development – III), neurological examination, Gross Motor Functional Classification System
5	El-Naggar 2018	Canada	Inclusion: singleton, preterm (24—30.6 weeks) Exclusion: monochorionic twins, major congenital anomalies, placental abruption, fetal anaemia, intention to withhold resuscitation	73 (Intervention: 37; Control: 36)	Cord was milked three times, speed 10 cm/s	Cord was clamped within 10 s of birth	Neonatal mortality, length of hospital stay
6	Erickson-Owens 2012	USA	Inclusion: singleton, term (> 37 weeks); caesarean delivery Exclusion: maternal medical and obstetric complications, severe anaemia, clotting disorders, suspected intrauterine growth restriction, smoking in pregnancy, non-English speaker, infant with confirmed diagnosis of intrauterine growth restriction, serious congenital anomalies	24 (Intervention: 12; Control: 12)	Cord was milked five times before clamping	Cord was clamped within 10 s of birth	Admission to NICU
7	Hosono 2007	Japan	Inclusion: singleton, preterm (24 – 28.6 weeks) and/or low birth weight (< 2500 g) Exclusion: multiple births, major congenital anomalies, chromosomal anomalies, hydrops fetalis	40 (Intervention: 20; Control: 20)	Cord was milked 2–3 times at 20 cm/s before being clamped	Cord was clamped immediately after birth	Neonatal mortality
8	Katheria 2014	USA	Inclusion: singleton, preterm (23 – 31.6 weeks) Exclusion: imminent delivery, monochorionic multiples, incarcerated mothers, placenta previa, concern for abruptions, refusal to perform the intervention by the obstetrician	60 (Intervention: 30; Control: 30)	Cord was milked at 20 cm over 2 s, repeated twice	Cord was clamped after 14 s (± 9 s)	Neonatal mortality
9	Katheria 2015	USA	Inclusion: singleton, preterm (23 – 31.6 weeks) Exclusion: monochorionic multiples, incarcerated mother, placenta previa, concern for abruption, Rh sensitisation, hydrops, congenital anomalies, obstetrician declined to perform the intervention	197 (Intervention: 75; Control: 79)	Cord was milked four times over 2 s, with a 1-2 s pause between milking, then clamped at 20 s after birth	Cord was clamped at least 45 s after birth (42 s ± 12 s)	Neonatal mortality
10	Katheria 2018	USA	Inclusion: singleton, Preterm (23 – 31.6 weeks) Exclusion: monochorionic multiples, incarcerated mothers, placenta previa, concern for placental abruption, Rh sensitization, hydrops, congenital anomalies	135 (Intervention: 70; Control: 65)	Cord was milked over 2 s and then repeated 3 additional times	Delayed cord clamping (45-60 s)	Neurodevelopmental outcomes at 22–26 months CA (Bayley Scales of Infant and Toddler development – III, Gross Motor Functional Classification System, neurological examination)
11	Krueger 2015	USA	Inclusion: singleton, preterm (22 – 31.6 weeks) Exclusion: fetal anomalies, suspected placental abruption	67 (Intervention: 35; Control: 32)	1/3 – 2/3 of the length of the umbilical cord was stripped between two fingers 4 times, with 4-5 s pause in between, and clamped after 30 s	Cord was clamped at 30 s after birth	Length of hospital stay, neonatal mortality
12	Kumawat 2022	India	Inclusion: term and late preterm (≥ 34 weeks) Exclusion: short umbilical cord (i.e., < 25 cm), prolapsed cord, abnormal cord and placenta, Rh-negative mothers, hydrops fetalis, delayed cry after birth, gross congenital malformations	168 (Intervention: 84; Control: 84)	Umbilical cord was cut at 30 s and milked three times at a speed of 10 cm/s	Cord was clamped at 30 s after birth	Admission to NICU
13	Mangla 2020	India	Inclusion: late preterm and term (35.0 – 42.6 weeks) Exclusion: fetal hydrops, major congenital malformation, Rh isoimmunization, new-borns born through meconium-stained liquor who were non-vigorous at birth, forceps or vacuum assisted delivery, new-borns born to HIV positive mother, maternal eclampsia	144 (Intervention: 72; Control: 72)	Umbilical cord was milked four times and clamped at 12.9 s (± 0.8 s)	Cord was clamped 60 s after birth	Admission to NICU
14	Mercer 2016	USA	Inclusion: singleton, preterm (24 – 31.6 weeks) Exclusion: multiple gestation, prenatally diagnosed major congenital anomalies, severe or multiple maternal illnesses, mothers who were at risk for loss to follow-up	161 (Intervention: 74; Control: 87)	Cord milked once at 30-45 s after birth	Cord was clamped within 10 s of birth	Neurodevelopmental outcomes at 18–22 months (Bayley Scales of Infant and Toddler development – III: Motor Score only)
15	Panburana 2020	Thailand	Inclusion: singleton, term (37—42 weeks) Exclusion: umbilical cord length less than 25 cm or cord abnormality(such as true knots or cord prolapse), multiple gestation, maternal Rh-negative blood group, positive anti-HIV, positive HBsAg, and syphilis infection during pregnancy, antenatal diagnosed major congenital anomalies of foetus or apparent at birth, fetal hydrops and fetal growth restriction, intrapartum fetal non reassuring or fetal distress, non-vigorous neonates, unstable maternal hemodynamic condition, placenta abruption, placenta previa, uterine rupture, declined to participate	168 (Intervention: 84; Control: 84)	Cord was milked 3 times, at 25 cm length, at 10 cm/s with 2 s interval and then clamped	Cord was clamped 60 s after birth	Length of hospital stay
16/17	Rabe 2011/ Rabe 2016	UK	Inclusion: singleton, preterm (24 – 32.6 weeks) Exclusion: multiple pregnancies (twins and more), fetal hydrops, rhesus sensitization, major congenital abnormalities	58 (Baseline -Intervention:; Control:) (2 year FU—Intervention: 22; Control: 17) (3.5 year FU—Intervention: 18; Control: 11)	Cord was milked four times at a speed of 20 cm/s	Cord was clamped at 30 s after birth	Neonatal mortality, length of hospital stay, glucose concentration (on admission), neurodevelopmental outcomes at 2 years and 3.5 years (Bayley Scales of Infant and Toddler development – III)
18	Shirk 2019	USA	Inclusion: singleton, preterm (23 – 34.6 weeks) Exclusion: major and minor congenital anomalies (not including trisomy markers), precipitous delivery that prevented completion of the protocol, placental abruption, uterine rupture, infants known to be at risk of anaemia, patient delivered at outside institution after random assignment. Once enrolled, a patient was excluded if they had a category 3 fetal heart rate tracing or prolonged fetal bradycardia	204 (Intervention: 100; Control: 104)	Milking / stripping of 20 cm of umbilical cord four times, allowing for refill between each milking manoeuvre	Cord was clamped at 60 s after birth	Neonatal mortality
19	Silahli 2018	Turkey	Inclusion: preterm (≤ 32 weeks) Exclusion: twin-to-twin transfusion syndrome, fetal and maternal bleeding, dysmorphic features, conotruncal heart disease	75 (Intervention: 38; Control: 37)	Cord was milked at 20 cm, 3 times before clamping	Cord was clamped within 10 s of delivery	Length of hospital stay
20	Song 2017	Korea	Inclusion: preterm (24 -32.6 weeks) Exclusion: multiple gestations, rhesus sensitization, fetal hydrops, major fetal anomalies, no consent provided	66 (Intervention: 34; Control: 32)	Cord was milked 4 times at 20 cm/s with a 2 s pause, which took approximately 15-20 s	Cord was clamped immediately after delivery	Length of hospital stay, neonatal mortality
21	Xie 2022	China	Inclusion: singleton, preterm (< 34 weeks) Exclusion: postpartum haemorrhage, major congenital anomalies, hydrops fetalis, haemolysis disease, multiple births, SGA infants	253 (Intervention: 121; Control: 132)	Cord was milked for 2 s, repeated four times	Cord was clamped immediately after birth	Neonatal mortality
**Delayed cord clamping**							
22	Andersson 2011	Sweden	Inclusion: singleton, vaginal delivery, term (37.0 – 41.6 weeks) Exclusions: serious congenital malformations, syndromes, other congenital diseases that could affect the outcome measures	344 (DCC:170; Control:174)	Cord was clamped at 180 s	Cord was clamped ≤ 10 s	Admission to NICU, Admission to NICU for hypoglycaemia
23	Andersson 2013	Sweden	Inclusion: singleton, vaginal delivery, term (> 37 weeks) Exclusion: serious congenital malformations, syndromes, other congenital diseases of the newborn infant that could affect the outcome measures	365 (DCC: 185; Control: 180)	Cord was clamped at 180 s	Cord was clamped ≤ 10 s	Neurodevelopmental outcomes at 4 months (Ages and Stages Questionnaire)
24	Andersson 2014	Sweden	Inclusion: singleton, vaginal delivery, term (> 37 weeks) Exclusion: serious congenital malformations, syndromes or other congenital diseases of the newborn infant that could affect the outcome measures	340 (DCC: 172; Control: 168)	Cord was clamped at 180 s	Cord was clamped ≤ 10 s	Neurodevelopmental outcomes at 12 months (Ages and Stages Questionnaire)
25	Andersson 2015	Sweden	Inclusion: vaginal delivery, term (37–41 weeks) Exclusion: serious congenital malformations, syndromes, other congenital diseases of the newborn infant that could affect the outcome measures	263 (DCC: 141; Control: 122)	Cord was clamped at 180 s	Cord was clamped ≤ 10 s	Neurodevelopmental outcomes at 48 months (Wechsler Preschool and Primary Scale of Intelligence-III; Ages and Stages Questionnaire -3)
26	Armanian 2017	Iran	Inclusion: preterm (< 34 weeks) Exclusion: non-admission to the NICU, twin pregnancy, attending clinician not compliant with the study protocol, parents’ refusal to participate, major congenital anomalies, asphyxia	60 (DCC: 30; Control: 30)	Cord was clamped 30-45 s after birth	Cord was clamped within 5-10 s	Neonatal mortality, length of hospital stay
27	Backes 2016	USA	Inclusion: singleton, preterm (22.5 – 27.6 weeks) Exclusion: placental abruption, placental previa, multiple gestations, chromosomal abnormalities (including trisomy 21), known major congenital malformations, attending obstetrician refusal to participate	40 (DCC: 18; Control 22)	Cord was clamped between 30-45 s	Cord was clamped within 10 s	Neonatal mortality, length of hospital stay
28	Berg 2021	Nepal	Inclusion: singleton, late preterm and term (34 – 41 weeks) Exclusion: clinical history of hypertension, infection, diabetes, any chronic medical condition	347 (DCC: 179; Control: 168)	Cord was clamped at 180 s	Cord was clamped at < 60 s	Neurodevelopmental outcomes at 3 years (Ages and Stages Questionnaire -3)
29	Cavallin 2019	Italy	Inclusion: singleton, elective caesarean section, term (> 39 weeks) Exclusion: multiple gestations, major congenital malformations and/or chromosomic abnormalities, intrauterine growth restriction and/or fetal hydrops, cord abnormalities (i.e., a length < 20 cm, funicular prolapse, or funicular knots)	80 (DCC: 40; Control: 40)	Cord was clamped > 60 s	Cord was clamped within 10 s	Glucose concentration (at birth)
30	Celikel 2022	Turkey	Inclusion: singleton, late term, term (36–42 weeks) Exclusion: chronic systemic disease, endocrine or metabolic disease during pregnancy, chronic drug or multivitamin use, fetal anomalies, multiple pregnancy, infants with suspected sepsis, anomalies, fetal distress, requiring postnatal resuscitation	60 (DCC: 28; Control (32)	Cord clamping was done at 60 s	Cord clamping was done within 10 s	Admission to NICU
31	Cernadas 2006	Argentina	Inclusion: singleton, term (> 37 weeks) Exclusion: clinical disease (diabetes, preeclampsia, hypertension), any other complications; congenital malformations or intrauterine growth restriction (estimated fetal weight < 10th percentile)	254 (DCC: 1 min: 83; 3 min: 83; Control: 88)	Two delayed clamping groups, 60 s (45-75 s) and 3 min (> 150 s)	Cord was clamped within 15-20 s	Admission to NICU, length of hospital stay
32	Chen 2018	China	Inclusion: singleton, term (37.0 – 41.6 weeks), birth weight 2500-400 g, vaginal delivery Exclusion: mothers refusal; congenital fetal anomalies; Apgar < 6 at 1 min, requirement for resuscitation and oxygen therapy, severe IUGR (< 3%), mothers who received cortisone, anticonvulsants, antidepressants, thyroid hormone, or insulin	720 (DCC: 90 in each group; Control: 90)	The cord was clamped at 30 s, 60 s, 90 s, 120 s and 150 s	Cord was clamped at < 15 s (11.8 ± 2.5 s)	Admission to NICU
33	Chopra 2018	India	Inclusion: low birth weight (< 25000 g) and late preterm (> 35 weeks) Exclusion: placental abruption or previa, congenital malformations, Rh isoimmunised, multiple pregnancies. Post randomization exclusion criteria: infants born at 10th centile, needing resuscitation, infant birth weight ≥ 10th percentile	113 (DCC: 55; Control: 58)	Cord was clamped > 60 s	Cord was clamped immediately	Incidence of hypoglycaemia (undefined), neonatal mortality
34	Das 2018	India	Inclusion: preterm (30.0–33.6 weeks) Exclusion: multiple pregnancies, major congenital malformation, hydrops fetalis	At 40 weeks = 390 (Intervention:193, Control: 197) At 9–12 months = 349 (Intervention: 171, Control: 178) At 24–30 months = 323 (Intervention: 158, Control: 165)	Cord was clamped at 60 s	Cord was clamped within 10 s	Neurodevelopmental outcomes at 40 weeks post-menstrual age (Amiel-Tison) and 9–12 months corrected age (Denver II) and 24–30 months chronological age (Developmental Assessment Scale for Indian Infants)
35	Datta 2017	India	Inclusion: singleton, preterm (34 – 36.6 weeks) Exclusion: gross congenital anomaly, hydrops, Rhesus negative pregnancy	Baseline: 117 FU: 112 (DCC: Baseline: 58, FU: 54; Control: Baseline:59, FU: 58)	Cord was clamped between 30-60 s	Cord was clamped within 20 s	Neurodevelopmental outcomes at day 1- and 37-weeks CA (Neurobehavioral Assessment of Preterm Infant: motor development score)
36	De Angelis 2022	Italy	Inclusion: singleton, vaginal delivery, term (37–41 weeks) Exclusion: multiple pregnancies, preterm delivery, induced labour, operative delivery, maternal hypertension, abnormal placentation, maternal bleeding disorders, planned cord blood banking	122 (DCC: 62; Control: 60)	Cord was clamped < 60 s after birth, or when pulsation stopped	Cord was clamped within 15 s	Neonatal mortality, admission to NICU
37	De Bernardo 2020	Italy	Inclusion: elective caesarean section, term (37–42 weeks), birth weight normal for gestational age Exclusion: pathologies, toxicomaniac, those who smoked or took drugs during pregnancy; admitted to NICU or needing resuscitation, new-borns that showed hypoxic-ischemic events: detachment of placenta, prolapse of the funiculus, uterine rupture, shoulder dystocia, premature rupture of fetal membranes, placenta previa, maternal collapse, embolism amniotic, maternal cardiac arrest, monochorionic twins, fetal hydrops, umbilical cord damaged, isoimmunization Rh, respiratory, malformative diseases	132 (DCC: 66; Control: 66)	Cord was clamped at 60 s	Cord was clamped immediately after birth	Glucose concentration (2 h after birth)
38	Digal 2021	India	Inclusion: singleton, IUGR, fetal weight < 10th percentile, preterm (≥ 28 weeks) Exclusion: hemodynamic instability, placenta previa/abruptio placentae, multiple gestation, Rh-negative blood group, major congenital malformation, fetal hydrops, requiring resuscitation at birth; GA < 28 weeks	110 (DCC: 55; Control: 55)	Cord was clamped after 60 s	Cord was clamped within 30 s	Admission to NICU, length of hospital stay
39/40	Duley 2017 / Armstrong-Buisseret 2019	UK	Inclusion: preterm (< 32 weeks) Exclusion: monochorionic twins; triplets or higher-order multiple pregnancy, major congenital malformation	270 (DCC: Baseline: 135; FU: 115; Control: Baseline: 135; FU:103)	Cord was clamped at ≥ 120 s	Cord was clamped within 20 s	Breastfeeding at discharge (undefined), neonatal mortality, length of hospital stay, neurodevelopmental outcomes at 2 years CA (Bayley Scales of Infant and Toddler development -III or Ages and Stages Questionnaire-3)
41	Feitosa 2021	Brazil	Inclusion: singleton, term (37–42 weeks), vaginal delivery Exclusion: High risk pregnancies, forceps delivery, resuscitation of neonate	580 (DCC: 278; Control: 282)	Cord clamping was done at 8 min (5 – 12.3 min), the umbilical cord was gently palpated every 30 s until pulsation stopped, allowing spontaneous drainage of blood from the placenta to the newborn	Cord remained intact and clamped at 180 s	Breastfeeding at discharge (exclusive), admission to NICU, length of hospital stay
42	Hemmati 2020	Iran	Inclusion: preterm (26 – 34 weeks) Exclusion: parent or clinician refusal, severe congenital anomalies, need for resuscitation, presence of placental abruption, placenta previa, clamping of the cord before or after the specified reference time intervals	148 (DCC: 69; Control: 79)	Cord was clamped between 30- 45 s	Cord was clamped after 10-15 s	Neonatal mortality, length of hospital stay
43	Hofmeyer 1988	South Africa	Inclusion: singleton, preterm (35 weeks)	38 (DCC: 24; Control: 14)	Cord was clamped 60 s after birth	Cord was clamped immediately	Neonatal mortality
44	Hofmeye, 1993	South Africa	Inclusion: low birth weight (< 2000 g)	86 (DCC: 40; Control: 46)	Cord was clamped 60-120 s after birth	Cord was clamped immediately	Neonatal mortality
45	Jomjak 2021	Thailand	Inclusion: singleton, moderate – late preterm (32–36.6 weeks) Exclusion: major severe congenital anomalies, chromosomal abnormalities, multifetal gestations, maternal coagulopathy, maternal anaemia, placenta previa, placenta abruption, fetal non-reassuring, fetal distress, non-vigorous neonate, denied participation	110 (DCC: 55, Control: 55)	Cord was clamped within 60 s	Cord was clamped within 5 s	Neonatal mortality, admission to NICU, length of hospital stay
46	Korkut 2019	Turkey	Inclusion: singleton, maternal diabetes (any), term (≥ 37 weeks) Exclusion: hydrops fetalis, major congenital anomaly, congenital infection, multiple gestation, no informed consent, any neonates whose birth was not attended by one of the researchers	80 (DCC: 40; Control: 40)	Cord was clamped at ≥ 60 s	Cord was clamped immediately after birth	Incidence of hypoglycaemia (defined as blood glucose levels of < 2.2 mmol/L in the first 4 h and < 2.5 mmol/L 3–24 h postnatally), severity of hypoglycaemia (severe hypoglycaemia defined as defined as blood glucose levels of < 1.4 mmol/L in the first 4 h and < 1.9 mmol/L 3–24 h postnatally), receipt of treatment for hypoglycaemia, admission to NICU
46	Krishnan 2015	India	Inclusion: singleton, vaginal delivery, term (> 37 weeks) Exclusion: pre-existing medical complications (heart disease, renal failure, other chronic illnesses); on any one of the following drugs (anticonvulsants, antidepressants, thyroid hormone, insulin, chemotherapy, or cortisone); infants anticipated to require resuscitation; major congenital anomalies; infants fed formula before obtaining ferritin levels at 6 weeks of age	76 (DCC: 37; Control: 39)	Cord was clamped 180 s	Cord was clamped 10 s after birth	Length of hospital stay
48	Kugelman 2007	Israel	Inclusion: preterm (24 – 34.6/7 weeks) Exclusion: parents refused consent; vaginal bleeding due to placenta previa or abruption or placental tear; major anomaly; severe intrauterine growth restriction (IUGR; < 3%); maternal gestational diabetes treated with insulin; suspected twins, twin transfusion syndrome or discordant twins; and maternal drug abuse	65 (DCC: 30; Control: 35)	Cord was clamped 30-45 s	Cord was clamped < 10 s	Neonatal mortality, glucose concentration (undefined timing—in delivery room), length of hospital stay
49	Mercer 2022	USA	Inclusion: singleton, term (37 – 41.6 weeks) Exclusion: medical or obstetrical complications (hypertension, pre-eclampsia, diabetes, smoking, substance abuse and suspected intrauterine growth restriction), infants with evidence of intrauterine growth restriction, serious congenital anomalies	41 (DCC: 21; Control: 20)	Cord was clamped at ≥ 5 min (if cord couldn’t be clamped it was milked 5 times before clamping)	Cord was clamped at < 20 s	Neurodevelopmental outcomes at 12 months (Mullen Scale of Early Learning; Brief Infant Toddler Social Emotional Assessment)
50	Mercer 2018	USA	Inclusion: singleton, term (37 – 41.6 weeks) Exclusion: medical or obstetrical complications (hypertension, pre-eclampsia, diabetes, smoking, substance abuse and suspected intrauterine growth restriction)	56 (DCC: 31; Control: 25)	Cord was clamped at > 5 min. If unable to delay the clamp, cord was milked 5 times before clamping. Clamp time was 172 s ± 188 s)	Cord was clamped < 20 s (28 s ± 7.6 s)	Neurodevelopmental outcomes at 4 months (Mullen Scales of Early learning)
51	Mercer 2017	USA	Inclusion: singleton, term (37 – 41.7 weeks) Exclusion: evidence of medical or obstetrical complications (hypertension, pre-eclampsia, diabetes, smoking, substance abuse and suspected intrauterine growth restriction), infants with evidence of intrauterine growth restriction, serious congenital anomalies	73 (DCC: 37; Control: 36)	Cord was clamped at > 5 min. If unable to delay the clamp, cord was milked 5 times before clamping	Cord was clamped < 20 s (23.1 s ± 5.9 s)	Breastfeeding at discharge (undefined)
52	Mercer 2010	USA	Inclusion: preterm (24 – 31.6 weeks) Exclusion: obstetrician’s refusal to participate, major congenital anomalies, multiple gestations, intent to withhold care, severe maternal illnesses, placenta abruption or previa	58 (DCC: 29; Control: 29)	Cord was clamped 30-45 s	Cord was clamped < 10 s	Neurodevelopmental outcomes (Bayley Scales of Infant and Toddler development -II) at 7.3 months CA
53	Mercer 2003	USA	Inclusion: singleton, preterm (24–31.6/7 weeks) Exclusion: obstetrician or parents refused consent, intent to withhold or withdraw care, placenta previa or abruption, maternal bleeding, major anomaly	32 (DCC: 16, Control: 16)	Cord was clamped 30-45 s	Cord was clamped 5-10 s	Incidence of hypoglycaemia (defined as blood glucose < 2.2 mmol/L in first 4 h postnatally), glucose concentration (within the first 12 h), length of hospital stay
54	Nouraie 2019	Iran	Inclusion: term (> 37 weeks) Exclusion: maternal complications (diabetes, cardiovascular, renal-pulmonary diseases, preeclampsia, placental abruption and polyhydramnios), mothers most recent delivery had not required the use of forceps or vacuum extractors and was not accompanied with complications such as haemorrhage, dystocia or prolonged labour, no history of known developmental (genetic) disorders or congenital anomalies in either parent families, preterm birth, Apgar score of ≥ 7, birth weight > 2.5 kg	400 (DCC: 200; Control: 200)	Cord was clamped between 90-120 s	Cord was clamped < 60 s	Neurodevelopmental outcomes at 4 months (Ages and Stages Questionnaire)
55	Oxford Midwives Research Group. 1991	UK	Inclusion: vaginal delivery, singleton, term (> 37 weeks) Exclusion: receiving medication other than iron and vitamin supplements; women whose baby was to be adopted; parents who had a specific preference for early or late cord clamping; babies who showed signs of stress in utero	552 (DCC: 296; Control: 256)	Cord was clamped 180 s after birth, or when pulsation stopped	Cord was clamped “as soon as possible” after birth	Breastfeeding at discharge (undefined)
56	Purisch 2019	USA	Inclusion: singleton, elective caesarean section, term (≥ 37.0 weeks) Exclusion: placenta previa, placenta abruption, prenatally diagnosed fetal anomalies, fetal anaemia, fetal growth restriction, preeclampsia, significant maternal anaemia, bleeding disorders, planned cord blood banking, refusal of blood products, women with caesarean deliveries scheduled on weekends or postponed to evening hours	113 (DCC: 57; Control: 56)	Cord was clamped at 60 s (63 s, IQR 61-65 s)	Cord was clamped within 15 s (6 s, IQR 5-8 s)	Admission to NICU
57	Rana 2019	Nepal	Inclusion: vaginal delivery, term (> 37 weeks) Exclusions: any complications	540 (DCC: 270; Control: 270)	Cord was clamped at ≥ 180 s	Cord was clamped at ≤ 60 s	Neurodevelopmental outcomes (Ages and Stages Questionnare-3) at 12 months CA
58	Rana 2018	India	Inclusion: preterm (< 34 weeks) Exclusion: known congenital malformations, serious maternal illnesses (severe preeclampsia or eclampsia, uncompensated heart disease, any abnormal bleeding before cord clamping), twins or triplets, and babies requiring immediate resuscitation at birth	100 (DCC: 50; Control: 50)	Cord was clamped after 120 s	Cord was clamped < 30 s	Length of hospital stay
59	Ranjit 2015	India	Inclusion: preterm (30 – 36.6 weeks) Exclusion: Rhesus negative blood group, monoamniotic/monochorionic twins, babies who did not receive the intervention due to need for resuscitation at birth	94 (DCC: 44; Control: 50)	Cord was clamped > 120 s	Cord was clamped immediately	Incidence of hypoglycaemia (undefined), neonatal mortality
60	Rashwan 2022	Egypt	Inclusion: singleton, assigned caesarean section, late term – term (36 – 38.6 weeks) Exclusion: intrapartum surgical complications such as uterine artery injury or lower segment extension, intrauterine fetal demise, medical disorders (anaemia, diabetes mellitus, abnormal placentation, placenta abruption, liquor abnormalities, or anomalous foetuses)	62 (DCC: 31; Control: 31)	Cord was clamped at 60 s	Cord was clamped within 15 s	Admission to NICU
61	Robledo 2022	Australia	Inclusion: preterm (< 30 weeks) Exclusion: fetal haemolytic disease, hydrops fetalis, twin transfusion, genetic syndromes, malformations	1419 (DCC: 709; Control: 710)	Cord was clamped at ≥ 60 s	Cord was clamped within 10 s	Neurodevelopmental outcomes at 2 years CA (Major disability as diagnosed by CP, vision loss, deafness, language problems; Ages and Stages Questionnaire-3)
62	Ruangkit 2019	Thailand	Inclusion: multiple gestations, preterm (28–36 weeks) Exclusion: diagnosed major congenital anomaly, twin-to-twin transfusion syndrome, twin anemic-polycythemic sequence, discordant twins (a weight difference of > 20%), neonatal mortality, hydrops, antepartum or intrapartum haemorrhage, when the medical care provider declined performing DCC	101 (DCC:51; Control: 50)	Cord was clamped at 30-60 s	Cord was clamped immediately (< 5 s)	Neonatal mortality, glucose concentration (on admission), length of hospital stay
63	Shao 2022	China	Inclusion: gestational diabetes, pre-diabetes and non-diabetic pregnancies, term (> 37 weeks) Exclusion: mothers with other pregnancy complications (hypertension disorders, intrahepatic cholestasis of pregnancy, maternal fever, multiple pregnancy, preterm labour, post-term pregnancy, emergency caesarean section, abnormal fetal presentation), birth weight < 2500 g, Apgar score of < 7, neonatal malformation, suspicious fetal distress, neonatal resuscitation, failed cord blood collection failed, missed blood gas parameters	441 (DCC: GDM:73, non-GDM: 107; Control: GDM:87, non-GDM:101)	Cord was clamped > 30 s	Cord was clamped < 15 s	Glucose concentration (within 15 min)
64	Shinohara 2021	Japan	Inclusion: singleton, vaginal delivery, term (> 37 weeks) Exclusion: maternal complications, fetal complications, emergency caesarean section, transferred to another hospital, not literate in Japanese, unable to return in 4 months	138 (DCC:68; Control: 70)	Cord was clamped at > 60 s or when pulsation stopped	Cord was clamped within 15 s	Breastfeeding at discharge (exclusive), neonatal mortality, ddmission to NICU
65	Soliman 2022	Egypt	Inclusion: term (> 37 weeks), elective caesarean Exclusion: history of inutero fetal distress, active resuscitation, twin or multiple gestation deliveries; major congenital anomalies, intrauterine growth restriction, perinatal asphyxia, perinatal hypoxic, ischemic event, Apgar score < 5 at 5 min, fetal umbilical artery pH < 7.0, and/or base deficit ≥ 16 mmol/L, presence of multisystem organ failure	68 (DCC:34; Control: 34)	Cord clamping was done at 120 s	Cord was clamped at 30 s	Glucose concentration (24 h after birth)
66	Songthamwat 2020	Thailand	Inclusion: singleton, vaginal delivery, term (37–41 weeks) Exclusion: severe medical complication (heart disease, chronic hypertension, or renal disease), fetal anomaly, fetal growth restriction, birth asphyxia, heavy bleeding immediately after birth, refusal to participate in the study	230 (DCC: 1 min: 76, 2 min: 77; Control: 77)	Two delayed clamping groups, 60 s and 120 s	Cord was clamped at 30 s	Admission to NICU
67	Songthamwat 2020b	Thailand	Inclusion: singleton, elective caesarean section, term (< 37 weeks) Exclusion: severe medical complication, fetal anomaly, fetal growth restriction, heavy bleeding immediately after birth, refusal to participate in this study, birth asphyxia, non-vigorous infant	159 (DCC: 80; Control: 79)	Cord was clamped at 60 s	Cord was clamped < 30 s	Admission to NICU
68	Tarnow-Mordi 2017	Australia	Inclusion: preterm (< 30 weeks) Exclusion: fetal haemolytic disease, hydrops fetalis, twin transfusion, genetic syndromes, malformations	1566 (DCC: 784; Control: 782)	Cord was clamped at ≥ 60 s	Cord was clamped ≤ 10 s	Neonatal mortality
69	Ultee 2007	The Netherlands	Inclusion: vaginal delivery, preterm (34.0 – 36.6 weeks) Exclusion: maternal overt diabetes or gestational diabetes, pregnancy-induced hypertension	37 (DCC: 18; Control: 19)	Cord was clamped within 180 s	Cord was clamped within 30 s (13.4 ± 5.6 s)	Incidence of hypoglycaemia (defined as < 2.0 mmol/L), glucose concentration (3 h after birth)
70	Vural 2018	Turkey	Inclusion: macrosomia (4000-4500 g), term (37 – 42 weeks) Exclusion: birth weight < 4000 g, need for resuscitation, < 37w or > 42w gestation, congenital heart disease, congenital malformations	51 (DCC: 25; Control: 26)	Cord clamping at 60 s after birth	Cord was clamped at 15 s after birth	Length of hospital stay
71	Yunis 2021	Egypt	Inclusion: preterm (< 34 weeks), mothers with antenatal diagnosis of placental insufficiency Exclusion: congenital anomaly, chromosomal anomaly, major resuscitation where delay of resuscitation was not possible	90 (DCC: 60; Control: 30)	Cord was clamped at 60 s	Cord was clamped within 10 s	Incidence of hypoglycaemia (defined by pre-feeding blood glucose level < 2.5 mmol/L), neonatal mortality, length of hospital stay

*Abbreviations*: *UCM* Umbilical cord milking, *DCM* Delayed cord milking, *S* seconds, *NICU* Neonatal intensive care unit, *CA* Corrected age, *IUGR* Intrauterine growth restriction, *GA* Gestational age, *CP* Cerebral palsy

Fifty studies compared DCC to control, of which 19 included preterm infants. Cord clamping delay varied from 30 s to 8 min. Twenty-two of these 50 studies were conducted in high-income countries (44%), 13 (26%) in upper-middle income countries, and 15 (30%) in low-middle income countries. They were conducted between 1988 and 2022, and sample size ranged from 32 to 1 566 infants (Table 1).

### Risk of bias in included studies

**Fig. 2:**
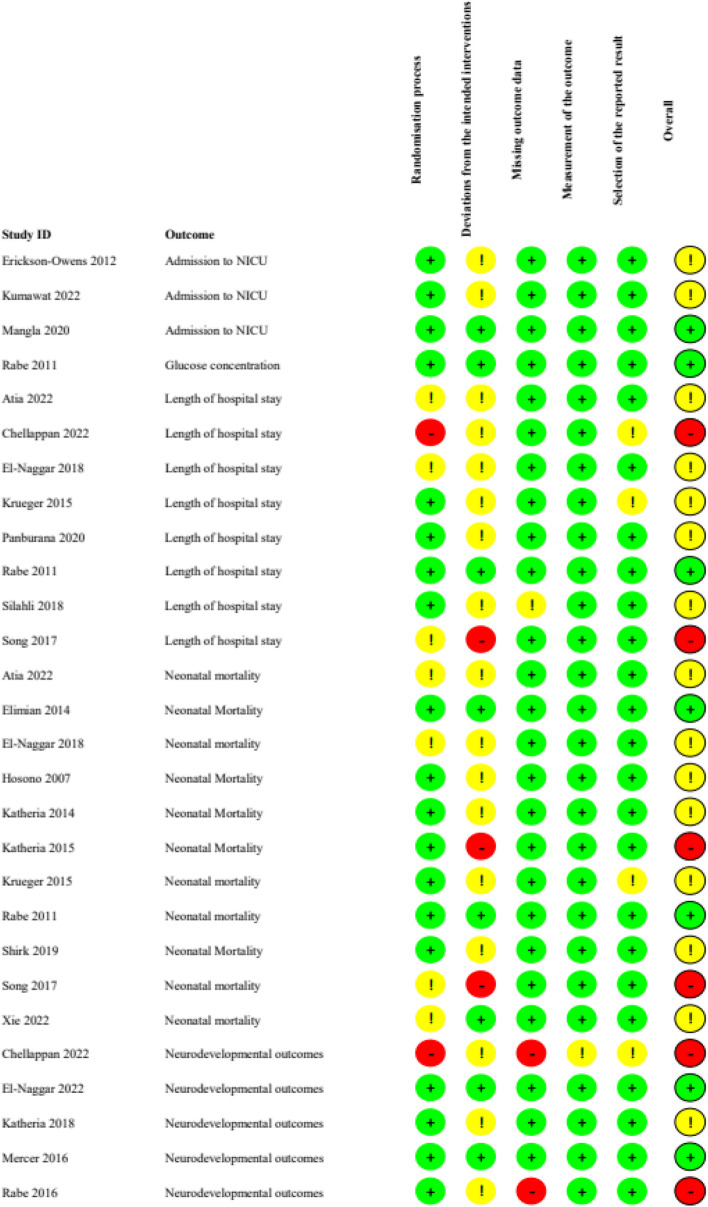
ROB-2 for umbilical cord milking outcomes

In the studies assessing UCM, high risk of bias was found in 2/8 studies looking at length of hospital stay outcome (25%), and some concerns were found in 5/8 studies (63%). For neonatal mortality, 18% of studies (2/11) showed high risk of bias, and 64% (7/11 studies) had some concerns. Two of the 5 studies (20%) assessing neurodevelopmental outcomes had high risk of bias, and 1/5 (20%) had some concerns. Only one study assessed glucose concentrations, and this was found to have low risk of bias (Fig. 2).

**Fig. 3:**
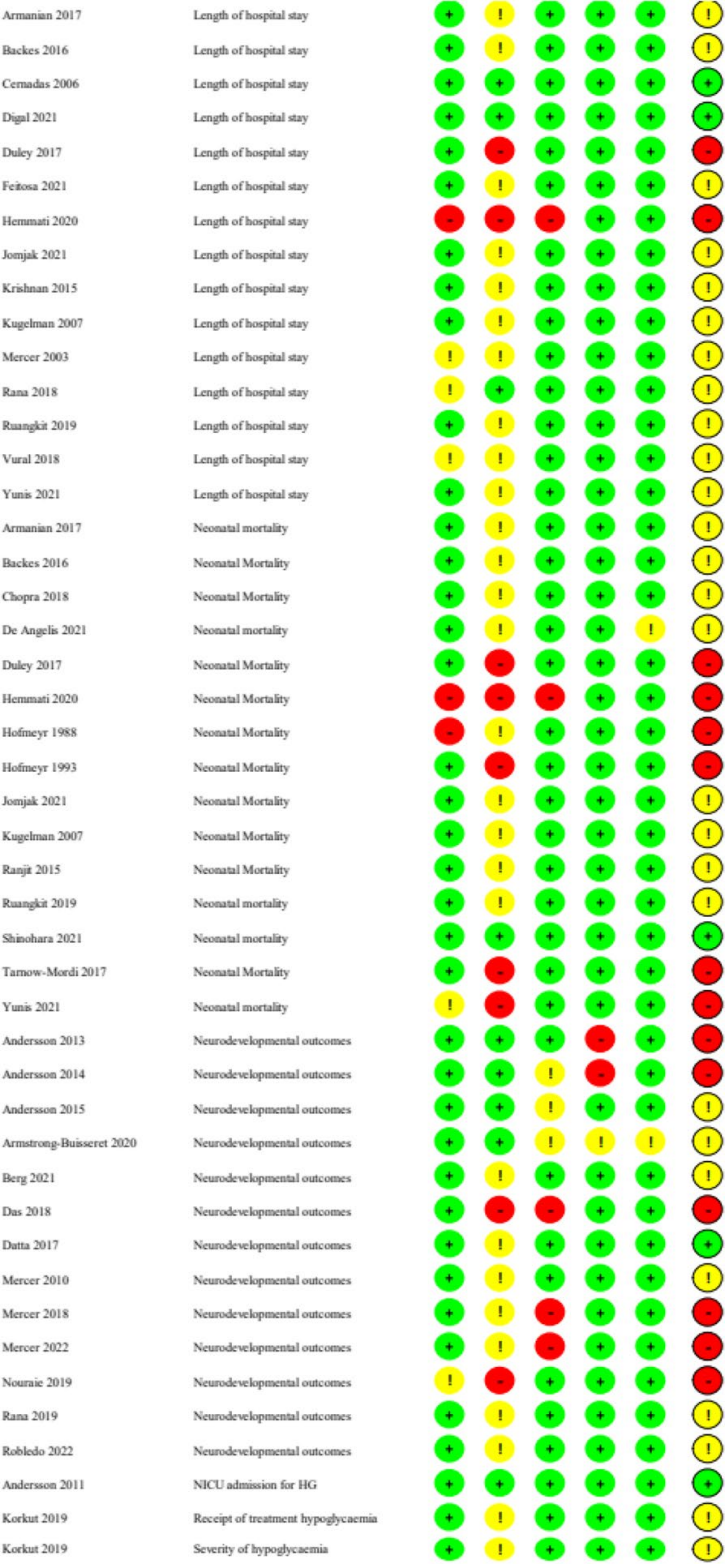
ROB-2 ROB-2 for delayed cord clamping outcomes

In the studies assessing DCC, one study assessing hypoglycaemia had high risk of bias, and 4/6 (67%) had some concerns (Fig. 3). For admission to NICU some studies (43%) had some concerns, as did many of the studies assessing breastfeeding at discharge (60%). For glucose concentrations, none of the studies had high risk of bias but most (75%) had some concerns, as did those assessing length of stay (73%). For studies assessing neonatal mortality, many had high risk of bias (40%) or some concerns (53%). For neurodevelopmental outcomes, many studies had high risk of bias (46%) or some concerns (46%).

### Outcomes

#### Umbilical cord milking

##### Primary outcome

**Hypoglycaemia**

No studies reported the incidence of hypoglycaemia.

**Blood glucose concentration**

One study [32] of 58 preterm neonates (24 0/7 – 32 6/7 weeks) found blood glucose concentration on admission to neonatal unit was 3.1 ± 1.5 mmol/l (*n* = 27) in the UCM group compared to 2.7 ± 1.4 mmol/l in the DCC group (31 infants) (Mean Difference (MD) = 0.40 (-0.35 to 1.15), *p* = 0.30).

##### Secondary outcomes

**Admission to neonatal intensive care unit**

**Fig. 4:**
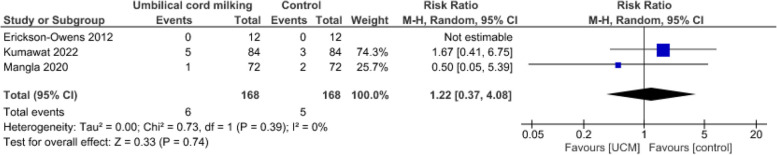
Effect of umbilical cord milking on admission to neonatal intensive care unit

The evidence suggests that UCM may result in little to no difference in admission to NICU (3 studies [33–35], 336 infants, RR = 1.22, CI:0.37–4.08, *p* = 0.74, I^2^ = 0%) (Fig. 4).

**Neurodevelopmental impairment**

**Fig. 5:**
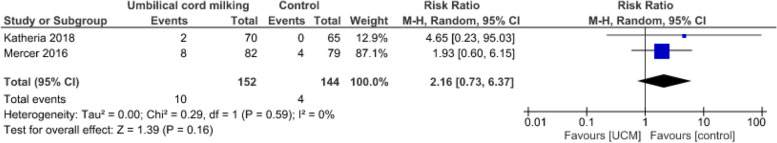
Effect of umbilical cord milking on neurodevelopmental impairment at 18-26 months follow up

Evidence from two studies [36, 37] suggests that UCM does not reduce the risk of neurodevelopmental impairment at 18–26 months (196 infants, RR = 2.16, CI:0.73 to 6.37, *p* = 0.16, I^2^ = 0%) (Fig. 5).

A further five studies assessed the effect of UCM on neurodevelopmental outcomes at various ages [36–40], but meta-analysis was not possible due to the heterogenous nature of the assessment methods and outcome interpretation. Of the five studies, one reported statistically significantly improved motor outcome after UCM at 18–22 months age [37]. The remaining four studies reported no difference in developmental outcomes between the intervention and control groups at 12 months [39], 22–26 months [36], 36 months [38] and 2 and 3.5 years [40].

**Neonatal mortality**

**Fig. 6:**
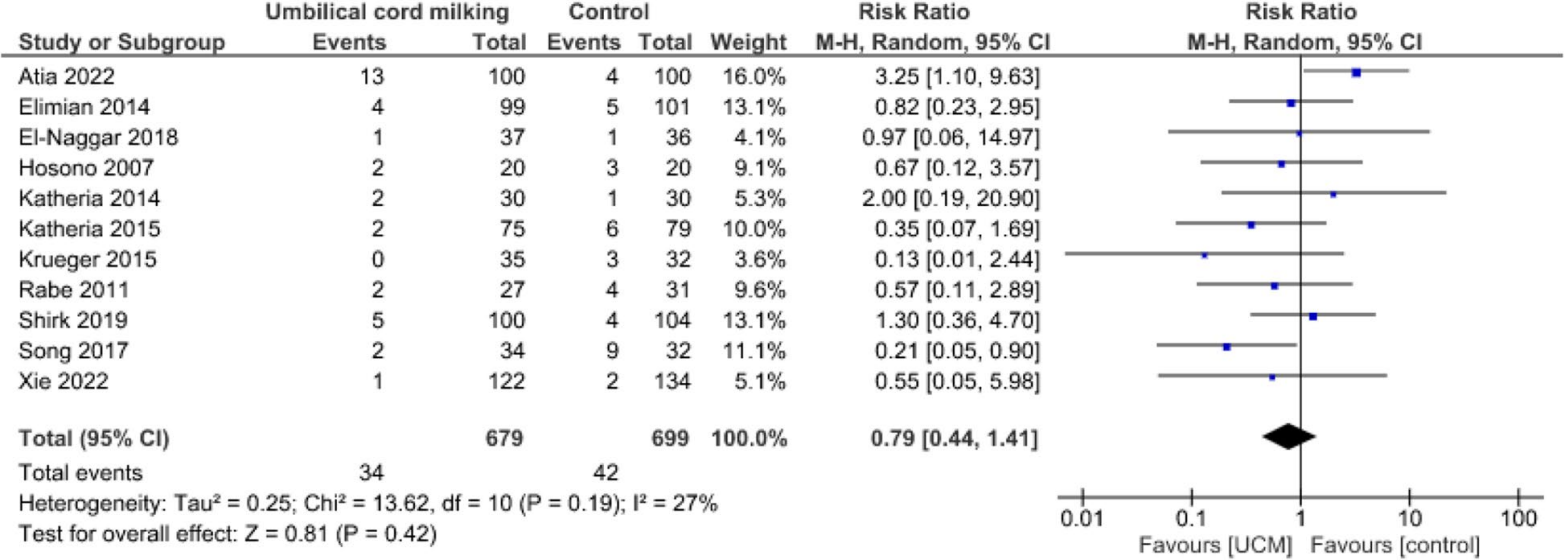
Effect of umbilical cord milking on neonatal mortality

In the 11 studies [32, 41–50] that reported neonatal mortality data, 76/1 378 infants died before discharge Fig. 6) The evidence suggests that UCM results in little to no difference in neonatal mortality (RR = 0.79, CI:0.44 to 1.41, *p* = 0.42, I^2^ = 27%).

**Length of hospital stay**

**Fig. 7:**
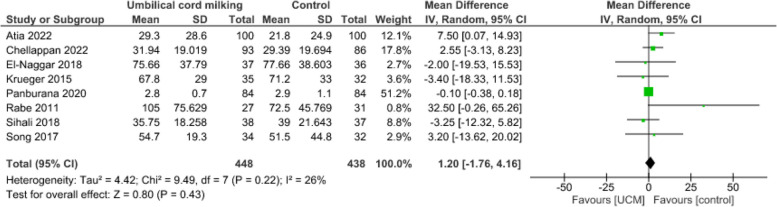
Effect of umbilical cord milking on length of hospital stay

Evidence from eight studies [32, 39, 41, 43, 47, 49, 51, 52] suggest that UCM may result in little to no difference in length of hospital stay (886 infants, MD = 1.20, CI: -1.76 to 4.16, *p* = 0.43, I^2^ = 26%, low certainty of evidence) (Fig. 7).

**Other outcomes**

There were no data available for the effect of UCM on breastfeeding at discharge, incidence of hypoglycaemia, receipt of treatment for hypoglycaemia during initial hospital stay, number of episodes of hypoglycaemia, severity of hypoglycaemia, hypoglycaemic injury on brain imaging, NICU admission for hypoglycaemia, cost of intervention or cost of neonatal care.

#### Delayed cord clamping

##### Primary outcome

**Incidence of hypoglycaemia**

**Fig. 8:**
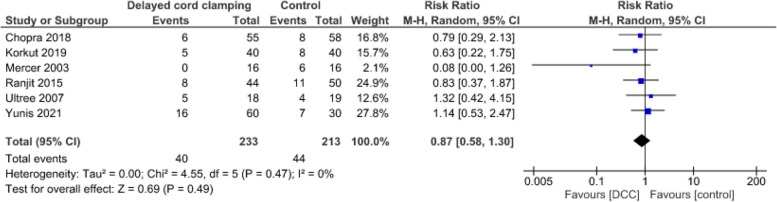
Effect of delayed cord clamping on incidence of neonatal hypoglycaemia

Evidence from six studies [53–58] suggests that DCC may result in little to no difference in neonatal hypoglycaemia (444 infants, RR = 0.87, CI:0.58 to 1.30, *p* = 0.49, I2 = 0%, low certainty of evidence) (Fig. 8). The definition of hypoglycaemia was not specified in two studies, blood glucose concentrations of < 2.2mmol/L in the first 4 h and/or < 2.5mmol/L at 3–24 h in two studies, < 2.0 mmol/L at 3 h in one study, and < 2.5mmol/L before a feed in one study. One study included term infants, one late preterm infant and four included preterm infants (Table 1).

**Blood glucose concentration**

**Fig. 9:**
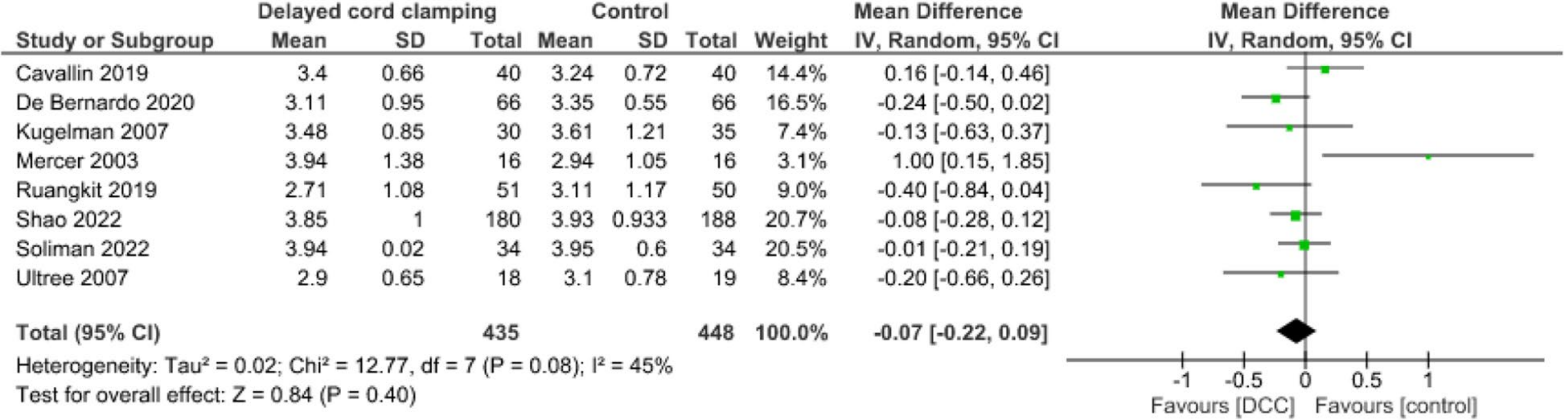
Effect of delayed cord clamping on blood glucose concentration

Evidence from eight studies [55, 57, 59–64] suggests that DCC may result in little to no difference in blood glucose concentrations during hospital stay (883 infants, MD = -0.07mmol/l, CI: -0.22 to 0.09, *p* = 0.40) (Fig. 9). Timing of blood glucose measurements varied between at birth and 24 h after birth (Table 1).

##### Secondary outcomes

**Admission to the neonatal intensive care unit**

**Fig. 10:**
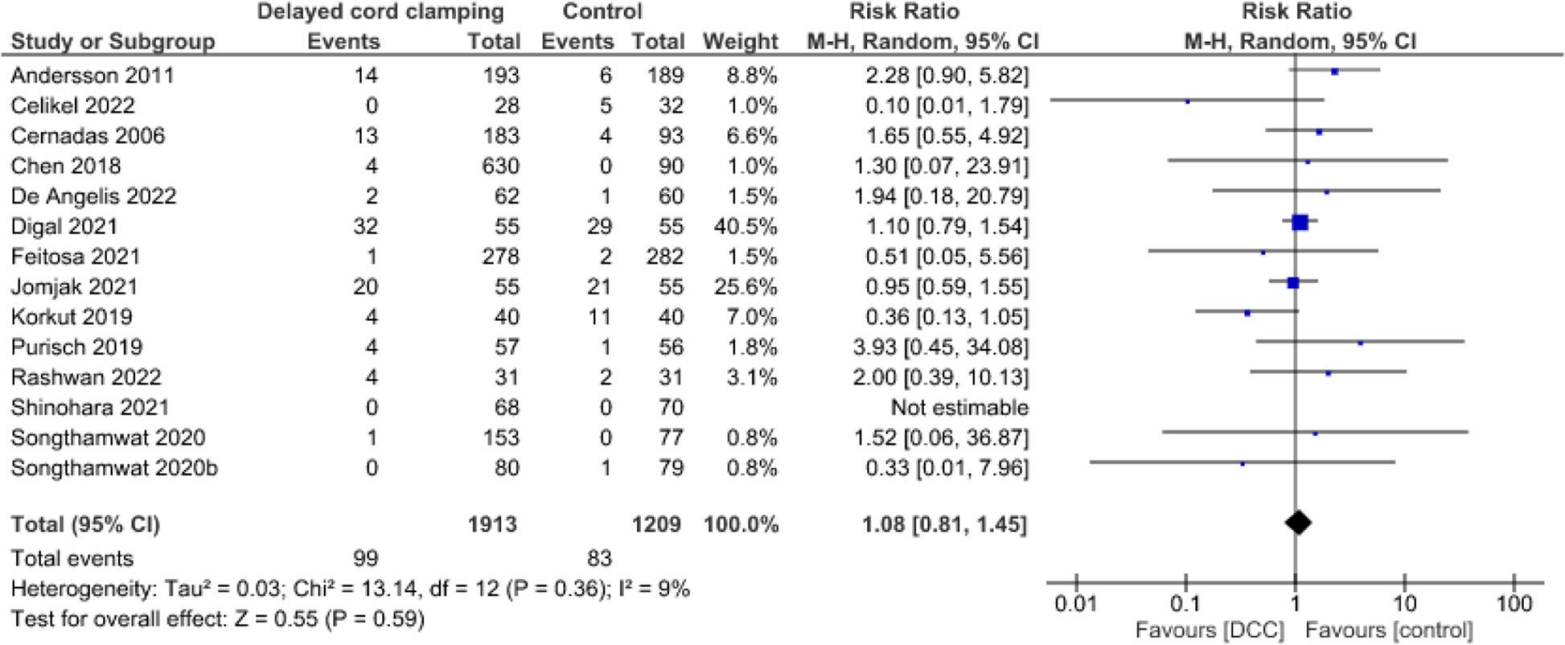
Effect of delayed cord clamping on admission to neonatal intensive care

Evidence from 14 studies [2, 54, 65–76] suggests that DCC may result in little to no difference in admission to NICU (3122 infants, RR = 1.08, CI: 0.81 to 1.45, *p* = 0.59, I^2^ = 9%) (Fig. 10).

**Admission to the neonatal intensive care unit for hypoglycaemia**

DCC may result in little to no difference in admission to NICU for hypoglycaemia (RR 1.95 (0.18, 21.35); *p* = 0.58). One study [65] of term infants (37 0/7 – 41 6/7 weeks gestation), compared DCC (≥ 180 s) to ECC (≤ 10 s) and found 2/174 infants (1.1%) from the DCC group were admitted to NICU due to hypoglycaemia, compared to 1/170 infants (0.6%) in the ECC group. This evidence was graded as low certainty.

**Receipt of treatment for hypoglycaemia during initial hospital stay**

DCC may not reduce the receipt of treatment for hypoglycaemia during initial hospital stay RR 0.14 (0.01, 2.68) *p* = 0.19. One study of women with gestational diabetes who gave birth to term infants (> 37 weeks gestation) [54] reported that no infants in the DCC group (cord clamping 60 s after birth, *n* = 40) required treatment (defined as glucose infusion) for hypoglycaemia, compared to three infants in the ECC group (cord clamped as early as possible, *n* = 40). This evidence was graded as low certainty.

**Severity of hypoglycaemia**

The same study [54] reported that 0/40 infants in the DCC group had severe hypoglycaemia (blood glucose < 1.4 mmol/l), compared to 2/40 (5%) in the ECC group. This evidence was graded as low certainty. DCC may not reduce incidence of severe hypoglycaemia RR 0.20 (0.01, 4.04); *p* = 0.29.

**Breastfeeding at discharge**

**Fig. 11:**
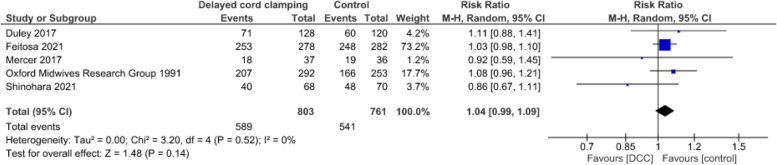
Effect of delayed cord clamping on breastfeeding at hospital discharge

DCC may result in little to no difference in breastfeeding at discharge (5 studies [70, 74, 77–79], 1 564 infants, RR = 1.04, CI:0.99 to 1.09, *p* = 0.14, I^2^ = 0%, low certainty evidence) (Fig. 11).

**Neurodevelopmental impairment**

**Fig. 12:**
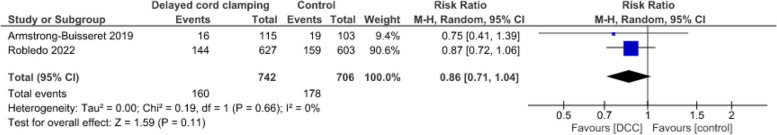
Effect of delayed cord clamping on neurodevelopmental outcomes at 12-24 months follow up

Data from two studies and 1448 infants[80, 81] suggest that DCC results in little to no difference in neurodevelopmental impairment at 12–24 months (RR = 0.86, CI:0.71–1.04, *p* = 0.11, I^2^ = 0%) (Fig. 12).

**Fig. 13:**
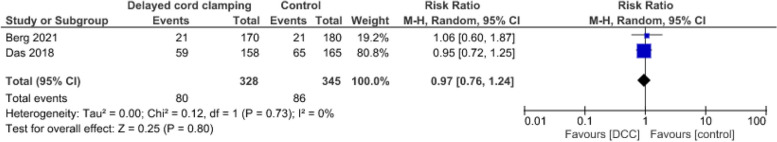
Effect of delayed cord clamping on neurodevelopmental outcomes at 24-48 months follow up

Similarly, evidence from two studies (673 infants) [82, 83] suggests that DCC results in little to no difference in neurodevelopmental impairment at 24–48 months (RR = 0.97, CI:0.76–1.24, *p* = 0.80, I^2^ = 0%) (Fig. 13).

A further twelve studies [82–92] reported the effect of DCC on neurodevelopmental outcomes, but the methods of assessment and outcome reporting differed between studies, making it difficult to conduct a meta-analysis. Of the 12 studies, five reported statistically significantly improved outcome with DCC, whilst six reported no difference, and one reported a reduced score for personal-social development with DCC compared to ECC (Table 2).

**Table 2:** Summary of neurodevelopmental outcomes

Reduced mild to moderate impairment with intervention	No significant difference in mild to moderate impairment with intervention	Increased mild to moderate impairment with intervention
Datta 2017 (37 weeks) – improved motor development-vigour and alert-orientation	Das 2018 (40 weeks, 9–12 months and 24–30 months)	Andersson 2013 (4 months) – reduced personal-social development
Andersson 2013 (4 months) – improved problem-solving	Mercer 2010 (7 months)	
Nouraie 2019 (4 months) – improved problem solving	Andersson 2014 (12 months)	
Rana 2018 (12 months) – improved in all domains except motor. Fewer infants in the DCC group were assessed to be at risk of having neurodevelopmental impairment	Mercer 2018 (4 months)	
Andersson 2015 (4 year) – improved fine motor and problem-solving	Mercer 2022 (12 months)	
	Berg 2021 (3 year)	

Figures in brackets are age at follow up assessment

**Neonatal mortality**

**Fig. 14:**
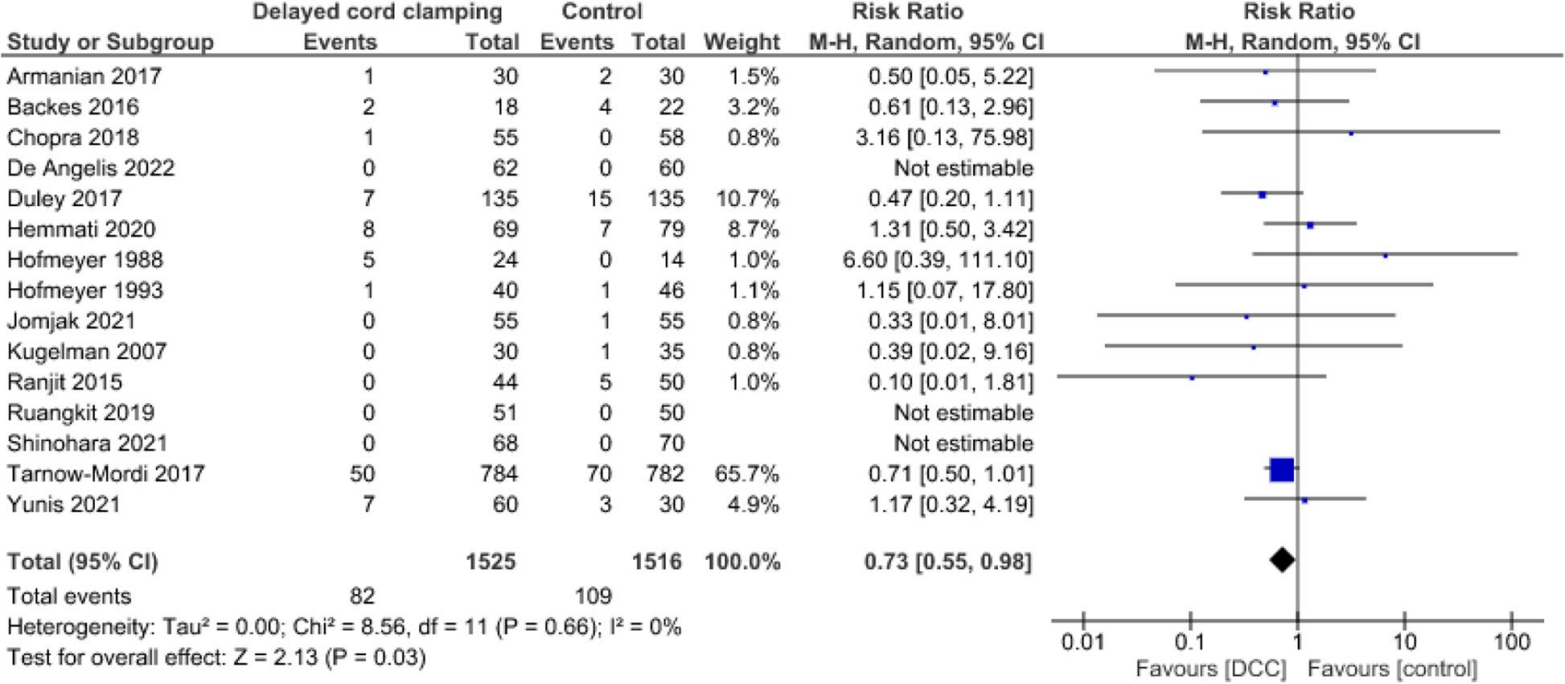
Effect of delayed cord clamping on neonatal mortality (at hospital discharge)

In the meta-analysis of 15 studies [2, 53, 56, 58, 61, 71, 74, 77, 93–98], a total of 191 infants out of 3 041 died before hospital discharge (Fig. 14). DCC probably results in a reduction in neonatal mortality (RR = 0.73, CI:0.55 to 0.98, *p* = 0.03, I^2^ = 0%).

**Length of hospital stay**

**Fig. 15:**
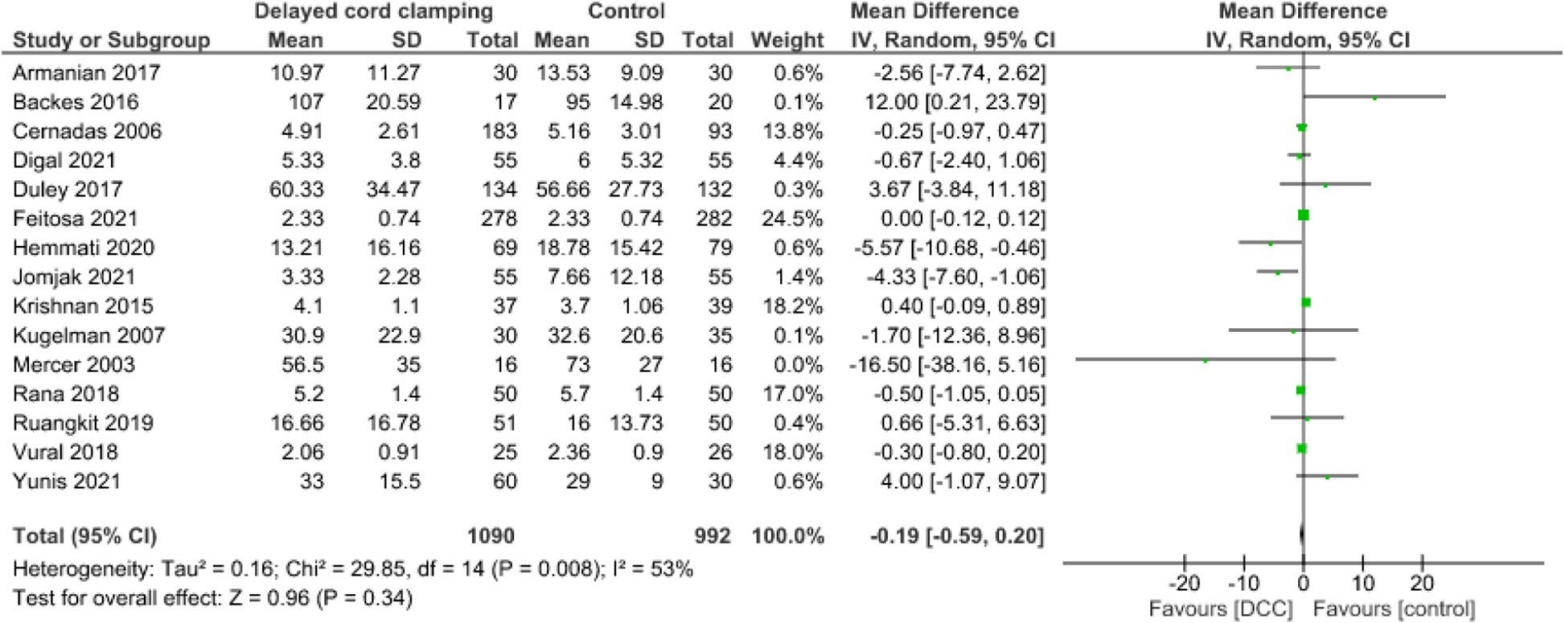
Effect of delayed cord clamping on length of hospital stay

Data from 15 studies [55, 58, 61, 62, 67, 69–71, 77, 89, 93–95, 99, 100] and 2 082 infants suggest that DCC results in little to no difference in length of hospital stay (MD=-0.19 days, CI:-0.59 to 0.20, *p*=0.34, I2=53%, low certainty evidence) (Fig. 15).

**Other outcomes**

No data were available for the effects of DCC on hypoglycaemic injury on brain imaging, cost of intervention and cost of neonatal care.

#### Subgroup analysis

In subgroup analyses for gestational age (term vs preterm infants), timing of the cord clamping (30–60 s vs > 60 s), mother’s diabetes status (yes/no), hospital setting, and delivery method (vaginal vs caesarean) there were no significant interactions between any of the subgroups and the available outcome variables (Appendix 2). There was insufficient data on risk factors for hypoglycaemia to conduct this pre-planned sub-group analysis.

#### Certainty of evidence (GRADE assessment)

For UCM, the certainty of evidence was assessed as low for length of hospital stay and was downgraded one level due to some concerns of risk of bias in most of the studies, and one level for wide 95% CI and relatively low sample size (Table 3). There were no data for the effect of UCM on the other GRADE outcomes.

**Table 3:** GRADE summary findings table for umbilical cord milking outcome/s

Outcomes	№ of participants (studies) Follow-up	Certainty of the evidence (GRADE)	Anticipated absolute effects	
			**Risk with Control**	**Risk difference with UCM**
Length of hospital stay	886 (8 RCTs)	⨁⨁◯◯ Low ^a^^,b^	The mean length of hospital stay was **45.7** days	MD **1.2 days longer** (1.76 fewer to 4.16 longer)

*CI* confidence interval, *MD* mean difference, *RR* risk ratio

Explanations

^a^Downgraded one level for risk of bias due to moderate risk of bias for this outcome

^b^Downgraded one level for imprecision due to wide CI and low sample size

For DCC, the certainty of evidence was low for all GRADE outcomes due to some concerns of risk of bias (neonatal hypoglycaemia, breastfeeding at discharge), and wide 95% CI or small sample size (neonatal hypoglycaemia and length of hospital stay). No significant publication bias was detected for length of stay outcome based on the funnel plot (Appendix 3). Admission to NICU for hypoglycaemia, severity of hypoglycaemia and receipt of treatment for hypoglycaemia were all rated as low certainty due to data coming from a single study (Table 4).

**Table 4:** GRADE summary findings table for delayed cord clamping outcomes

Outcomes	№ of participants (studies) Follow-up	Certainty of the evidence (GRADE)	Relative effect (95% CI)	Anticipated absolute effects	
				**Risk with ECC**	**Risk difference with DCC**
Neonatal hypoglycaemia	446 (6 RCTs)	⨁⨁◯◯ Low^a,b^	**RR 0.87** (0.58 to 1.30)	207 per 1,000	**27 fewer per 1,000** (87 fewer to 62 more)
Length of hospital stay	2082 (16 RCTs)	⨁⨁◯◯ Low^c,d^	-	The mean length of hospital stay was **24.5** days	MD **0.19 days shorter** (0.59 lower to 9.07 higher)
Breastfeeding at discharge	1564 (5 RCTs)	⨁⨁◯◯ Low^a,b^	**RR 1.04** (0.99 to 1.09)	711 per 1,000	**28 more per 1,000** (7 fewer to 64 more)
NICU Admission for hypoglycaemia	344 (1 RCT)	⨁⨁◯◯ Low^e^	RR 1.95 (0.18, 21.35)	6 per 1,000	**6 fewer per 1,000** (6 fewer to 6 fewer)
Severe hypoglycaemia	80 (1 RCT)	⨁⨁◯◯ Low^e^	RR 0.20 (0.01, 4.04)	50 per 1,000	**50 fewer per 1,000** (50 fewer to 50 fewer)
Receipt of treatment for hypoglycaemia	80 (1 RCT)	⨁⨁◯◯ Low^e^	RR 0.14 (0.01, 2.68)	75 per 1,000	**75 fewer per 1,000** (75 fewer to 75 fewer)

*CI* confidence interval, *DCC* Delayed cord clamping, *ECC* Early cord clamping, *MD* mean difference, *NICU* Neonatal intensive care unit, *RCT* Randomised controlled trial, *RR* risk ratio

Explanations

^a^Downgraded one level for risk of bias due to moderate risk of bias for this outcome

^b^Downgraded one level for imprecision due to wide CI and low sample size

^c^Downgraded one level for heterogeneity (large I^2^ and low *p*-value)

^d^Downgraded one level for imprecision due to wide CI

^e^Downgraded two levels for imprecision due to small sample size and only one study

## Discussion

### Summary of main results

The two main placental transfusion strategies to improve red blood cell volume after birth are DCC and UCM. This systematic review included a total of 71 studies and data from 14 268 infants. Despite including more studies than all reviews to date [15, 17, 19, 101, 102], we found no evidence for the effect of UCM on incidence of hypoglycaemia, and only one small study showing no significant difference in blood glucose concentrations between UCM and DCC groups [32]. In line with findings from previous reviews, we also found no significant differences in UCM compared to control groups for neonatal mortality or length of hospital stay [19, 101, 102], and no difference in risk of neurological impairment. However, data from large, well-designed studies for hypoglycaemia outcomes are lacking.

The benefits of DCC are well known, and delaying the cord clamp by 60–120 s is recommended as best practice in preterm and term infants [103]. To the best of our knowledge, this is the first systematic review to assess the effects of DCC on neonatal hypoglycaemia. We found that DCC may have little to no effect on the incidence of hypoglycaemia or blood glucose concentration, or on rate of NICU admission and breastfeeding at discharge. We found low certainty evidence from one study [65] that DCC may result in little to no difference in admission to NICU for hypoglycaemia compared to ECC. Data from another study showed that DCC may not effect receipt of treatment for hypoglycaemia and or severity of hypoglycaemia, compared with ECC [54].

There is evidence from several systematic reviews that DCC improves haemoglobin, iron levels and initial arterial blood pressure as well as reducing the risk of IVH and need for resuscitation compared to ECC [6, 8, 11, 104–106]. These effects also suggest that DCC has the potential to reduce hypoglycaemia, since many neonatal problems, including the need for resuscitation, hypotension and IVH, result in increased tissue glucose consumption. These effects also suggest that DCC may improve neurodevelopmental outcomes [107], However, we found no evidence that DCC altered either of these outcomes, possibly due to very limited data and substantial heterogeneity in study design.

This meta-analysis showed that DCC may reduce neonatal mortality (low certainty of evidence). This is in line with findings from studies of very preterm infants [108] and many similar reviews of preterm infants [104, 105], as well as a recent Cochrane review of evidence in preterm infants (average RR: 0.73, 95% CI: 0.54 to 0.98, moderate certainty) [11]. We also found no difference in length of hospital stay when comparing DCC with ECC (low quality of evidence). Although few systematic reviews to date have assessed this outcome, Li et al. [104] found that DCC reduced hospital stay by 3.79 days (95% CI = -4.16 to -3.42) compared to ECC. Their review included four studies of preterm infants only, which may account for the difference in findings.

### Overall completeness and applicability of the evidence

Although this is the first study to synthesise the evidence for UCM and DCC and neonatal hypoglycaemia, there are several gaps in the data available for this review. Firstly, no data were found for the effect of UCM on our primary outcome of neonatal hypoglycaemia, and data were lacking for several other pre-specified secondary outcomes. Secondly, there was large heterogeneity in the intervention (UCM varied from 2–5 times for 10-20cm/s) and control (varied from ECC to DCC after 60 s) designs.

For DCC, six studies (444 infants) reported neonatal hypoglycaemia as an outcome, but the evidence was rated as low certainty due to concerns of bias and imprecision. There was considerable variation in the DCC (30 s to 8 min) versus control (immediately to 180 s) timing. However, subgroup analysis of timing of the cord clamping showed no significant interaction between the different timings.

Optimal timing for screening blood glucose is uncertain, and in our review, for both UCM and DCC studies, the timing of the measurement of blood glucose concentrations differed. For example, for the UCM study glucose was measured on admission, whilst for the DCC studies some measurements were taken at birth and others within the first 24 h. Since glucose concentrations change rapidly within the first few hours after birth [5, 109], the timing of blood glucose concentration measurements may have contributed to variability in the findings. Likewise, measurement of neurological outcomes differed considerably among the studies, making synthesis and meta-analysis of the data challenging. In addition, only one study assessed the effects of DCC on neonatal hypoglycaemia outcomes such as severity, admission to NICU, and treatment received. This review also excluded studies of non-vigorous infants, and those requiring resuscitation, therefore the evidence may not be generalisable to this population.

### Quality of the evidence

The certainty of evidence was graded as low for all specified outcomes. As with most placental transition interventions [11], blinding the clinicians to the allocated intervention is not possible, although some studies did blind the outcome measurement. For many of the UCM studies, the sample sizes were small leading to imprecision. Similarly, many of the studies comparing the incidence of hypoglycaemia between DCC and ECC groups had small sample sizes. For many other neonatal hypoglycaemia-related outcomes, data were only available from one study.

### Quality of the review

To the best of our knowledge, this is the first review to investigate the impact of UCM and DCC on neonatal hypoglycaemia as a primary outcome. The large number of RCTs included in the review, more than any other review of UCM or DCC, is a key strength. However, the review does have certain limitations. Firstly, no data were found for the effects of UCM on incidence of hypoglycaemia, and only one study reported blood glucose concentrations. There was more evidence for the effects of DCC, with six studies assessing incidence of hypoglycaemia and eight studies measuring blood glucose concentrations. However, sample sizes were small, and the CIs were relatively large, therefore the results need to be interpreted with caution. Secondly, although there is potential for bias within the review process, we did take steps to minimise this by using at least two authors to independently screen, extraction, and assessment of quality. A third author was included for any discrepancies.

## Conclusion

Data are lacking from large, well-designed studies assessing the effects of various placental transfusion strategies on neonatal hypoglycaemia. We found no studies assessing the effects of UCM on neonatal hypoglycaemia, and no evidence that DCC altered the incidence of neonatal hypoglycaemia compared to ECC. Although there are many other benefits of UCM and DCC, more high-quality studies are needed to enable reliable conclusions about their effect on hypoglycaemia.

### Supplementary Information

**Supplementary Material 1.**

**Supplementary Material 2.**

**Supplementary Material 3.**

## Data Availability

Data access requests are to be submitted to the Data Access Committee via researchhub@auckland.ac.nz. Data will be shared with researchers with a sound proposal on reasonable request.

## Abbreviations

DCC: Delayed cord clamping
ECC: Early cord clamping
GRADE: Grading of Recommendations, Assessment, Development and Evaluation
IVH: Intraventricular haemorrhage
MD: Mean Difference
NICU: Neonatal intensive care unit
PRISMA: Preferred Reporting Items for Systematic Reviews and Meta-analysis
PROSPERO: Prospective register of systematic reviews
RCTs: Randomised controlled trials
RoB-2: Risk of Bias tool
RR: Risk Ratio
SD: Standard Deviation
UCM: Umbilical cord milking
