# Supplementary material for: Umbilical cord milking and delayed cord clamping for the prevention of neonatal hypoglycaemia: a systematic review and meta-analysis
Source: BMC Pregnancy Childbirth. 2024 Apr 8;24:248. doi: 10.1186/s12884-024-06427-w (PMC11000397; doi:10.1186/s12884-024-06427-w)
Supplement: Supplementary file 1 — Supplementary Material 1. [file 12884_2024_6427_MOESM1_ESM.docx]

**Supplementary Table 1. Search terms and results**

| **Database** | **#** | **Search strategy** | **Results from September 2022** |
| --- | --- | --- | --- |
| **Medline** | 1 | exp Umbilical Cord/ | 28,786 |
|  | 2 | "umbilical cord*".ab,kf,ti. | 27,324 |
|  | 3 | "clamp*".ab,kf,ti. | 95,772 |
|  | 4 | (placenta* adj2 transfus*).ti,ab,kw. | 329 |
|  | 5 | (perinatal adj2 transfus*).ti,ab,kw. | 47 |
|  | 6 | (fetal adj2 transfus*).ti,ab,kw. | 673 |
|  | 7 | ((umbilical or cord) adj5 (cut* or sever* or clamp* or delay* or early or tim* or milk* or Strip* or defer*)).mp. | 15,109 |
|  | 8 | exp Infant, Newborn/ or exp Infant/ | 1,229,100 |
|  | 9 | (babe or babes or baby* or babies or infant? or infantile or infancy or low birth weight or low birthweight or neonat* or neo-nat* or newborn* or new born? or newly born or premature or pre-mature or pre-matures or prematures or prematurity or pre-maturity or preterm or preterms or pre term? or preemie or preemies or premies or premie or VLBW or VLBWI or VLBW-I or VLBWs or LBW or LBWI or LBWs or ELBW or ELBWI or ELBWs or NICU or NICUs).ti,ab,kw. | 949,626 |
|  | 10 | 1 or 2 | 47,682 |
|  | 11 | 3 and 10 | 1,325 |
|  | 12 | 4 or 5 or 6 or 7 or 11 | 16,131 |
|  | 13 | 8 or 9 | 1,662,854 |
|  | 14 | 12 and 13 | 4,160 |
|  | 15 | randomized controlled trial.pt. | 577,072 |
|  | 16 | controlled clinical trial.pt. | 95,028 |
|  | 17 | randomized.ab,pt. | 575,690 |
|  | 18 | placebo.ab,pt. | 231,689 |
|  | 19 | clinical trials as topic.pt,sh. | 200,377 |
|  | 20 | randomly.ab,pt. | 391,260 |
|  | 21 | trial.pt,ti. | 270,299 |
|  | 22 | randomi?ed.ti,ab. | 743,954 |
|  | 23 | randomly.ti,ab. | 392,161 |
|  | 24 | trial.ti,ab. | 715,615 |
|  | 25 | 15 or 16 or 17 or 18 or 19 or 20 or 21 or 22 or 23 or 24 | 1,738,050 |
|  | 26 | exp animals/ not humans.sh. | 5,046,186 |
|  | 27 | 25 not 26 | 1,597,344 |
|  | 28 | 14 and 27 | 601 |
|  |  |  | **Results from March 2023** |
|  | 29 | limit 28 to last year | 64 |
|  |  |  | **665** |
| **Embase** | **#** | **Search strategy** | **Results from September 2022** |
|  | 1 | umbilical cord.mp. or exp umbilical cord/ | 82,948 |
|  | 2 | exp umbilical cord clamping/ or exp delayed cord clamping/ or exp early cord clamping/ | 593 |
|  | 3 | infant/ or exp newborn/ | 1,017,829 |
|  | 4 | placental transfusion.ab,kf,ti. | 258 |
|  | 5 | (milk* or strip*).ab,kf,ti. | 238,919 |
|  | 6 | "tim*".ab,kf,ti. | 5,843,153 |
|  | 7 | "cut*".ab,kf,ti. | 648,209 |
|  | 8 | "sever*".ab,kf,ti. | 4,277,175 |
|  | 9 | 5 or 6 or 7 or 8 | 9,809,620 |
|  | 10 | 1 and 9 | 29,421 |
|  | 11 | 2 or 4 or 10 | 29,870 |
|  | 12 | (infant, newborn or newborn or neonate or neonatal or premature or very low birth weight or low birth weight or VLBW or LBW or Newborn or infan* or neonat*).ab,kf,ti. | 998,653 |
|  | 13 | 3 or 12 | 1,481,057 |
|  | 14 | 11 and 13 | 11,209 |
|  | 15 | (randomised controlled trial or controlled clinical trial or randomised or placebo or clinical trials as topic or randomly or trial or clinical trial).ab,kf,ti. | 1,684,545 |
|  | 16 | 14 and 15 | 1,055 |
|  | 17 | limit 16 to human | 1,007 |
|  |  |  | **Results from March 2023** |
|  | 18 | limit 17 to last year | 100 |
|  |  |  | **1107** |
| **CINAHL** | # | **Search strategy** | **Results from March 2023** |
|  | S1 | (MH "Umbilical Cord+") | 4,465 |
|  | S2 | (MH "Umbilical Cord Clamping") | 257 |
|  | S3 | TI ( Clamp* OR Milk* OR Strip* OR Cut* OR Tim*) OR AB ( Clamp* OR Milk* OR Strip* OR Cut* OR Tim*) | 942,522 |
|  | S4 | S1 AND S3 | 1,106 |
|  | S5 | S2 OR S4 | 1,340 |
|  | S6 | (MH "Infant+") OR (MH "Infant, Very Low Birth Weight") OR (MH "Infant, Premature") OR (MH "Infant, Postmature") | 286,801 |
|  | S7 | (MH "Randomized Controlled Trials+") | 132,938 |
|  | S8 | (MH "Clinical Trials+") OR (MH "Clinical Trial Registry") | 343,899 |
|  | S9 | S7 OR S8 | 343,899 |
|  | S10 | (S7 OR S8) AND (S5 AND S6 AND S9) | **142** |
| **CENTRAL** | # | **Search strategy** | **Results from March 2023** |
|  | #1 | MeSH descriptor: [Umbilical Cord] explode all trees | 653 |
|  | #2 | MeSH descriptor: [Umbilical Cord Clamping] explode all trees | 7 |
|  | #3 | "milk*":ti,ab,kw or "strip*":ti,ab,kw or "cut*":ti,ab,kw or "tim*":ti,ab,kw | 25461 |
|  | #4 | "placental transfusion":ti,ab,kw | 115 |
|  | #5 | #1 and #3 | 27 |
|  | #6 | #2 or #5 or #4 | 142 |
|  | #7 | randomized controlled trial:pt or controlled clinical trial:pt or clinical trial:pt | 667742 |
|  | #8 | #6 and #7 | 61 |
|  | #9 | MeSH descriptor: [Infant] explode all trees | 35105 |
|  | #10 | #8 and #9 | **55** |
| **ICTRP** |  | **Search strategy** | **Results from March 2023** |
|  |  | cord AND clamp | 4 |
|  |  | cord and clamping | 31 |
|  |  | cord AND milking | 9 |
|  |  | cord AND stripping | 0 |
|  |  |  | **44** |
| **ClinicalTrials.gov / Current Controlled Trials** |  | **Search strategy** | **Results from March 2023** |
|  |  | Advanced search |  |
|  |  | Interventional studies \| cord clamping | 157 |
|  |  | Interventional studies \| cord milking | 45 |
|  |  | Interventional studies \| cord stripping | 7 |
|  |  |  | **209** |
| **ANZCTR** |  | **Search strategy** | **Results from March 2023** |
|  |  | Advanced search |  |
|  |  | Interventional studies \| cord clamping | 12 |
|  |  | Interventional studies \| cord milking | 1 |
|  |  | Interventional studies \| cord stripping | 0 |
|  |  |  | **13** |
