# Supplementary material for: Umbilical cord milking and delayed cord clamping for the prevention of neonatal hypoglycaemia: a systematic review and meta-analysis
Source: BMC Pregnancy Childbirth. 2024 Apr 8;24:248. doi: 10.1186/s12884-024-06427-w (PMC11000397; doi:10.1186/s12884-024-06427-w)
Supplement: Supplementary file 2 — Supplementary Material 2. [file 12884_2024_6427_MOESM2_ESM.docx]

**Supplementary Table 2: Subgroup analysis**

| **Umbilical cord milking Summary of subgroup analysis** | | | | | | |
| --- | --- | --- | --- | --- | --- | --- |
| **Outcomes** | **Subgroups** | **No. of participants (studies)** | **Risk ratio (RR) or mean difference (MD) (95% CI)** | **P for overall effect** | **I^2^** | **P for subgroup interaction** |
| Length of hospital stay | Term infants (≥37 weeks GA) | 168 (1 study) | -0.10 (-0.38-0.18) | 0.48 | N/A | 0.25 |
|  | Preterm infants (<37 weeks GA) | 718 (7 studies) | 2.54 (-1.93 -7.00) | 0.27 | 18% |  |

| **Delayed cord clamping Summary of subgroup analysis** | | | | | | |
| --- | --- | --- | --- | --- | --- | --- |
| **Outcomes** | **Subgroups** | **No. of participants (studies)** | **Risk ratio (RR) or mean difference (MD) (95% CI)** | **P for overall effect** | **I^2^** | **P for subgroup interaction** |
| Admission to NICU | Clamp timing 30-60 seconds | 1 233 (9 studies) | 1.06 (0.71-1.60) | 0.76 | 0% | 0.88 |
|  | Clamp timing >60 seconds | 2 059 (8 studies) | 1.12 (0.65-1.91) | 0.68 | 38% |  |
| Neonatal mortality | Clamp timing 30-60 seconds | 974 (10 studies) | 0.99 (0.57- 1.73) | 0.98 | 0% | 0.23 |
|  | Clamp timing >60 seconds | 2 267 (6 studies) | 0.67 (0.49-0.92) | 0.01 | 0% |  |
| Length of hospital stay | Clamp timing 30-60 seconds | 878 (10 studies) | -0.39 (-2.32 – 1.53) | 0.69 | 78% | 0.85 |
|  | Clamp timing >60 seconds | 1 297 (6 studies) | -0.60 (-1.46 – 0.27) | 0.18 | 92% |  |
| Glucose concentration | Clamp timing 30-60 seconds | 698 (5 studies) | -0.11 (-0.36 – 0.14) | 0.38 | 56% | 0.41 |
|  | Clamp timing >60 seconds | 185 (3 studies) | 0.01 (-0.14-0.17) | 0.86 | 0% |  |
| Incidence of hypoglycaemia | Clamp timing 30-60 seconds | 122 (2 studies) | 0.40 (0.02 -6.55) | 0.52 | 74% | 0.61 |
|  | Clamp timing >60 seconds | 324 (4 studies) | 0.84 (0.51-1.36) | 0.47 | 0% |  |
| Breastfeeding at discharge | Term infants (≥37 weeks GA) | 1 316 (4 studies) | 1.03 (0.98- 1.09) | 0.18 | 0% | 0.57 |
|  | Preterm infants (<37 weeks GA) | 148 (1 study) | 1.11 (0.88- 1.41) | 0.39 | N/A |  |
| Admission to NICU | Term infants (≥37 weeks GA) | 2 902 (12 studies) | 1.15 (0.63- 2.08) | 0.66 | 22% | 0.80 |
|  | Preterm infants (<37 weeks GA) | 220 (2 studies) | 1.05 (0.80- 1.39) | 0.72 | 0% |  |
| Neonatal mortality | Term infants (≥37 weeks GA) | 260 (2 studies) | N/A* | N/A | N/A | N/A |
|  | Preterm infants (<37 weeks GA) | 2 781 (13 studies) | 0.73 (0.55- 0.98) | 0.03 | 0% |  |
| Length of hospital stay | Term infants (≥37 weeks GA) | 963 (4 studies) | -0.00 (-0.24- 0.23) | 0.97 | 33% | 0.30 |
|  | Preterm infants (<37 weeks GA) | 1 119 (11 studies) | -0.89 (-2.54- 0.76) | 0.29 | 51% |  |
| Glucose concentration | Term infants (≥37 weeks GA) | 648 (4 studies) | -0.05 (-0.19- 0.08) | 0.46 | 26% | 0.97 |
|  | Preterm infants (<37 weeks GA) | 235 (4 studies) | -0.04 (-0.49- 0.40) | 0.85 | 64% |  |
| Incidence of hypoglycaemia | Term infants (≥37 weeks GA) | 80 (1 study) | 0.63 (0.22- 1.75) | 0.37 | N/A | 0.50 |
|  | Preterm infants (<37 weeks GA) | 366 (5 studies) | 0.92 (0.58- 1.45) | 0.71 | 3% |  |
| Neurological impairment (24-48 months) | Term infants (≥37 weeks GA) | 350 (1 study) | 1.06 (0.60- 1.87) | 0.84 | N/A | 0.73 |
|  | Preterm infants (<37 weeks GA) | 323 (1 study) | 0.95 (0.72-1.25) | 0.70 | N/A |  |
| Glucose concentration | Mothers with GDM | 160 (1 study) | 0.00 (-0.33- 0.33) | 1.00 | N/A | 0.67 |
|  | Mothers without GDM | 723 (8 studies) | -0.08 (-0.25- 0.09) | 0.34 | 48% |  |
| Breastfeeding at discharge | Hospital birth setting | 1 353 (3 studies) | 1.05 (1.00- 1.10) | 0.07 | 0% | 0.12 |
|  | Clinic birth setting | 111 (2 studies) | 0.87 (0.70- 1.09) | 0.23 | 0% |  |
| Neonatal mortality | Hospital birth setting | 2 781 (13 studies) | 0.73 (0.55- 0.98) | 0.03 | 0% | N/A |
|  | Clinic birth setting | 260 (2 studies) | N/A* | N/A | N/A |  |
| Breastfeeding at discharge | Vaginal delivery only | 1 243 (3 studies) | 1.03 (0.96- 1.11) | 0.36 | 24% | 0.78 |
|  | Any delivery | 321 (2 studies) | 1.07 (0.86- 1.32) | 0.55 | 0% |  |
| Admission to NICU | Vaginal delivery only | 2 232 (7 studies) | 1.02 (0.43- 2.39) | 0.97 | 31% | 0.64 |
|  | Caesarean delivery only | 334 (3 studies) | 1.91 (0.57- 6.34) | 0.29 | 0% |  |
|  | Any delivery | 556 (4 studies) | 1.05 (0.76- 1.46) | 0.76 | 15% |  |
| Neonatal mortality | Vaginal delivery only | 260 (2 studies) | N/A* | N/A | N/A | N/A |
|  | Any delivery | 2 781 (13 studies) | 0.73 (0.55- 0.98) | 0.03 | 0% |  |
| Length of hospital stay | Vaginal delivery only | 736 (3 studies) | -0.01 (-0.38- 0.36) | 0.95 | 65% | 0.26 |
|  | Any delivery | 1 346 (12 studies) | -0.61 (-1.60- 0.38) | 0.22 | 48% |  |
| Glucose concentration | Vaginal delivery only | 37 (1 study) | -0.20 (-0.66- 0.26) | 0.40 | N/A | 0.81 |
|  | Caesarean delivery only | 280 (3 studies) | -0.04 (-0.24-0.17) | 0.73 | 49% |  |
|  | Any delivery | 566 (4 studies) | -0.04 (-0.40- 0.33) | 0.84 | 64% |  |
| Incidence of hypoglycaemia | Vaginal delivery only | 37 (1 study) | 1.32 (0.42- 4.15) | 0.64 | N/A | 0.44 |
|  | Any delivery | 409 (5 studies) | 0.82 (0.53- 1.26) | 0.36 | 0% |  |

*RR not estimable due to lack of events in both groups.
