# Supplementary figures and images for: Umbilical cord milking and delayed cord clamping for the prevention of neonatal hypoglycaemia: a systematic review and meta-analysis
Source: BMC Pregnancy Childbirth. 2024 Apr 8;24:248. doi: 10.1186/s12884-024-06427-w (PMC11000397; doi:10.1186/s12884-024-06427-w)

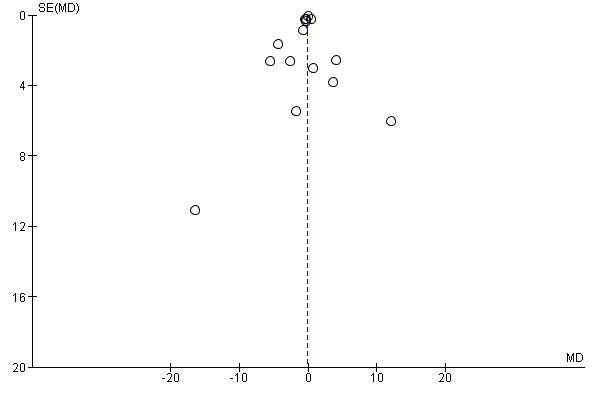


Supplementary Figure 1 Funnel plot comparison for DCC: Outcome: length of hospital stay

Supplement: Supplementary file 3 — Supplementary Material 3. [file 12884_2024_6427_MOESM3_ESM.docx]
